# GC-MS Profiling, Antioxidant and Enzyme Inhibitory Activities of Basil (*Ocimum basilicum* L.) and Clove (*Syzygium aromaticum* L.) Essential Oils and Their Binary Mixtures

**DOI:** 10.3390/molecules31183292

**Published:** 2026-09-17

**Authors:** Hubert Sytykiewicz, Iwona Łukasik, Sylwia Goławska

**Affiliations:** Faculty of Natural Sciences, Institute of Biological Sciences, University of Siedlce, 14 Prusa St., 08-110 Siedlce, Poland; iwona.lukasik@uws.edu.pl (I.Ł.); sylwia.golawska@uws.edu.pl (S.G.)

**Keywords:** essential oils, antioxidant activity, cyclooxygenase-2 inhibition, collagenase inhibition, *Ocimum basilicum*, *Syzygium aromaticum*

## Abstract

Combining essential oils (EOs) with contrasting chemical profiles may result in biological effects that cannot be reliably predicted from the properties of the individual oils. This study evaluated the chemical composition, biological activities, and interactions of basil (*Ocimum basilicum* L.; EOB) and clove (*Syzygium aromaticum* L.; EOC) essential oils. Gas chromatography–mass spectrometry (GC-MS) identified 41 volatile constituents in EOB and 29 in EOC. EOB was dominated by estragole (73.0%) and methyl eugenol (7.2%), whereas EOC was characterized mainly by eugenol (55.0%) and (E)-β-caryophyllene (32.9%). The oils were tested individually over a concentration range of 2.5–500 µg/mL, while binary EOB:EOC mixtures (1:1, 1:2, and 2:1; *v*/*v*) were evaluated at total essential oil concentrations of 5, 25, and 50 µg/mL. Antioxidant activity was assessed using linoleic acid peroxidation and ABTS^•+^ radical scavenging assays, whereas enzyme-inhibitory activity was evaluated against cyclooxygenase-2 (COX-2) and collagenase. EOC showed stronger ABTS^•+^ radical scavenging activity and greater inhibition of COX-2 and collagenase, while EOB exhibited greater inhibition of lipid peroxidation. Bliss independence analysis revealed concentration- and ratio-dependent interactions. Synergy (ΔBliss > +10 percentage points) was observed for the 1:2 EOB:EOC mixture at 5 and 25 µg/mL in the COX-2, collagenase, and ABTS^•+^ assays, and for all three mixture ratios at 5 µg/mL in the lipid peroxidation assay; no antagonistic interactions were detected. These findings demonstrate that the biological activity and interaction profiles of basil–clove essential oil mixtures depend on both mixture composition and concentration. Further studies in cell-based and in vivo models are required to confirm their efficacy, mechanisms of action, and safety.

## 1. Introduction

Skin aging is a complex biological process shaped by intrinsic and extrinsic factors, including senescence, ultraviolet (UV) radiation, environmental pollutants, and oxidative stress [1]. A central mechanism behind cutaneous aging is the excessive formation of reactive oxygen species (ROS), which disturb redox balance and cause oxidative damage to lipids, proteins, and nucleic acids [2]. Oxidative alteration of cellular components, especially membrane lipids and structural proteins, leads to weakened skin barrier function, disrupted cellular signalling, and gradual loss of tissue integrity. These changes accelerate extracellular matrix (ECM) breakdown, reduce dermal strength, and result in wrinkle formation, decreased elasticity, and chronic inflammatory skin conditions [3,4,5]. In addition, chronic low-grade inflammation is recognized as an important factor in skin aging and degenerative dermatological diseases. ROS-driven activation of pro-inflammatory signalling pathways increases the expression of cyclooxygenase-2 (COX-2), which promotes prostaglandin production and intensifies inflammatory responses. Moreover, oxidative stress triggers the activation of matrix metalloproteinases (MMPs), particularly collagenases, leading to ongoing degradation of collagen fibres, a defining feature of both intrinsic and photoinduced skin aging [6,7,8,9]. Therefore, coordinated regulation of oxidative stress, inflammatory mechanisms, and collagen-degrading enzymes provides a basis for developing agents directed at several processes associated with skin aging.

Plant-derived EOs have attracted growing interest because their chemically diverse constituents may exert antioxidant, anti-inflammatory, antimicrobial, and enzyme-modulating effects. Essential oils are complex mixtures of volatile secondary metabolites, mainly terpenoids and phenylpropanoids, and their biological activities may arise from both individual constituents and interactions among multiple components. These interactions may modify the overall biological response of an essential oil mixture, depending on its chemical composition, the relative proportions of its constituents, and the experimental system used. Genetic background, environmental conditions, geographical origin, and extraction procedures strongly influence essential oil composition and activity [10,11,12].

*Ocimum basilicum* L. (Lamiaceae) is a widely distributed aromatic plant characterized by substantial chemical polymorphism and diverse essential oil chemotypes, including estragole-, linalool-, methyl eugenol-, and eugenol-dominant profiles. Basil essential oil composition varies with genotype, environmental conditions, developmental stage, and geographical origin, resulting in considerable differences in biological properties among cultivars [13,14,15]. Basil essential oil has shown antioxidant and anti-inflammatory potential; however, its influence on specific molecular targets linked to skin aging, such as collagen-degrading and inflammatory enzymes, remains less well defined. *Syzygium aromaticum* L. (Myrtaceae), commonly known as clove, is an aromatic plant whose essential oil is predominantly obtained from floral buds and has been widely investigated for its biological activity. Clove essential oil typically contains high levels of eugenol, along with β-caryophyllene, eugenyl acetate, and other volatile compounds. Clove essential oil has well-documented antioxidant, anti-inflammatory, antimicrobial, and enzyme-modulating properties [16,17]. Recent studies have increasingly examined botanicals as agents that may act on several processes associated with skin aging. Herbal cosmeceuticals are regarded as potential alternatives to synthetic compounds due to their broad biological effects and potential to act on several mechanisms involved in skin deterioration [6,10,18]. However, most studies have focused on single plant extracts or individual essential oils, whereas quantitative evaluation of essential oil combinations remains limited. Although basil and clove essential oils have been investigated individually, their interactions in terms of antioxidant activity, COX-2 inhibition, and collagenase inhibition remain insufficiently characterized. Quantitative assessment of essential oil combinations is therefore important to determine whether their combined effects are consistent with synergistic interaction, show no substantial deviation from Bliss independence, or are consistent with antagonistic interaction [19,20,21], particularly because essential oils are chemically complex mixtures containing numerous potentially interacting constituents [7,22,23].

To the best of our knowledge, the interactions between basil and clove essential oils have not been systematically characterized across multiple mixture ratios and concentrations using a quantitative Bliss independence approach. The present study therefore combined GC-MS chemical profiling, enzyme inhibition assays targeting COX-2 and collagenase, antioxidant assays (linoleic acid peroxidation inhibition and ABTS^•+^ radical scavenging), and quantitative interaction analysis based on the Bliss independence model. The objectives of this study were to: (i) identify the chemical composition of basil and clove essential oils by gas chromatography-mass spectrometry (GC-MS); (ii) determine their inhibitory activity against COX-2 and collagenase; (iii) assess their antioxidant capacity through linoleic acid peroxidation inhibition and ABTS^•+^ radical scavenging assays; and (iv) quantitatively characterize the interactions within binary EOB:EOC mixtures relative to the Bliss independence model and determine whether the observed responses were consistent with synergistic interaction, showed no substantial deviation from Bliss independence, or were consistent with antagonistic interaction.

## 2. Results and Discussion

### 2.1. GC-MS Characterization of the Examined Essential Oils

**Figure 1:**
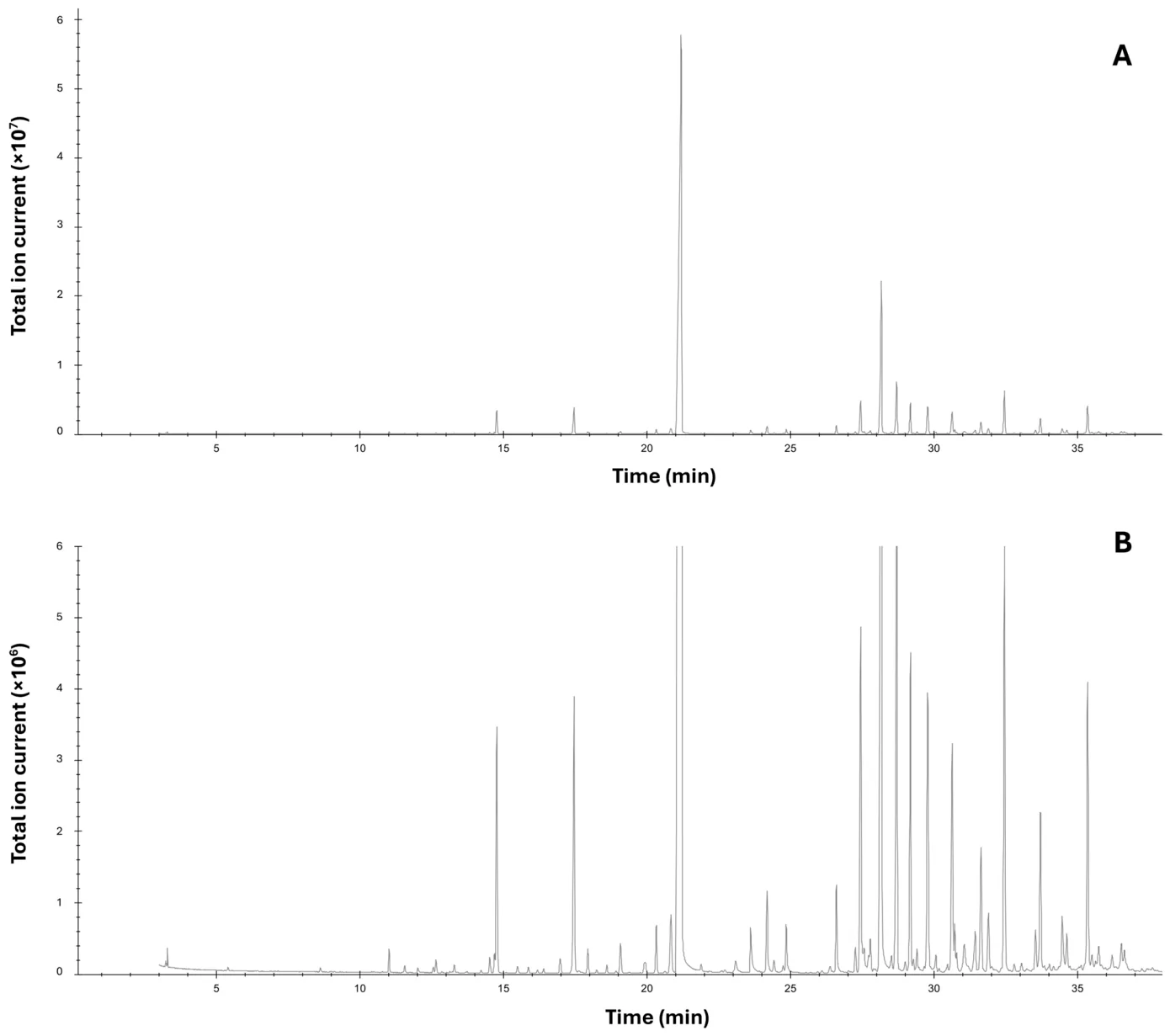
Total ion chromatogram (TIC) of basil essential oil acquired by GC-MS in full-scan mode. (**A**) Full-scale TIC showing the complete total ion-current intensity range. (**B**) The same TIC displayed using an expanded low-intensity scale to facilitate visualization of minor chromatographic signals; the most intense peaks exceed the displayed intensity range in panel **B**.

**Figure 2:**
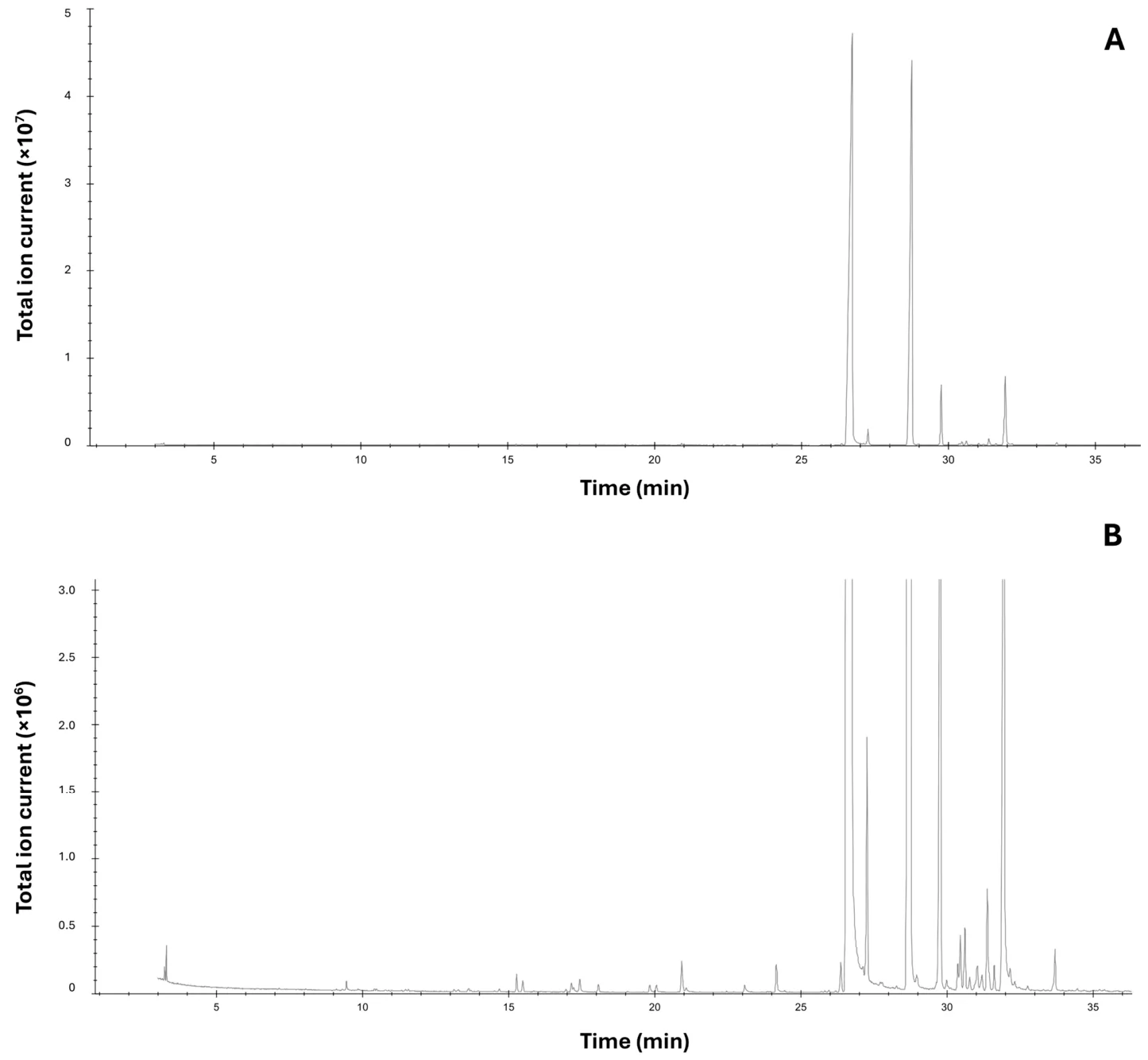
Total ion chromatogram (TIC) of clove essential oil acquired by GC-MS in full-scan mode. (**A**) Full-scale TIC showing the complete total ion-current intensity range. (**B**) The same TIC displayed using an expanded low-intensity scale to facilitate visualization of minor chromatographic signals; the most intense peaks exceed the displayed intensity range in panel **B**.

The gas chromatography-mass spectrometry (GC-MS) analysis identified 41 volatile constituents in the basil essential oil (EOB) and 29 volatile constituents in clove essential oil (EOC), accounting for 99.2% and 99.8% of the respective volatile profiles (Table 1 and Table 2, Figure 1 and Figure 2).

Basil essential oil exhibited an estragole-dominant profile, with estragole as the principal constituent (73.0%), followed by methyl eugenol (7.2%). Oxygenated monoterpenes, including 1,8-cineole and linalool, as well as sesquiterpene hydrocarbons such as (E)-β-caryophyllene and α-humulene, were detected in minor amounts. This composition is consistent with previously reported estragole-rich chemotypes of *Ocimum basilicum* L. originating from different geographical regions, where considerable variability in the relative abundance of phenylpropanoids and terpenoids has been observed [13,24,25]. The clove essential oil exhibited a eugenol-dominant chemotype, with eugenol identified as the major constituent (55.0%). The second most abundant compound was (E)-β-caryophyllene (32.9%), followed by eugenyl acetate (4.7%) and α-humulene (3.5%). This profile agrees with previously described compositions of clove essential oils, in which eugenol is typically the predominant phenylpropanoid accompanied by sesquiterpene hydrocarbons, particularly β-caryophyllene [16,17,26]. Although both essential oils contained phenylpropanoid-derived compounds, their qualitative and quantitative profiles differed considerably. Basil essential oil was characterized by a predominant estragole-based composition with minor contributions from oxygenated monoterpenes and sesquiterpenes, whereas clove essential oil showed a characteristic eugenol/β-caryophyllene profile. These differences may reflect species-specific regulation of secondary metabolism, resulting in distinct phenylpropanoid and terpenoid patterns characteristic of basil and clove essential oils [25,27]. Differences in the qualitative and quantitative composition of essential oils are commonly associated with species-specific metabolic characteristics as well as genetic background, environmental conditions, geographical origin, plant developmental stage, and extraction procedures [27,28,29]. From a chemical classification perspective, basil essential oil was enriched mainly in phenylpropanoids, with estragole representing the dominant compound, whereas clove essential oil displayed a characteristic predominance of eugenol together with a substantial sesquiterpene fraction. These differences illustrate the distinct volatile profiles of the two plant species and provide a chemical basis for their differentiation at the compositional level. Notably, the high abundance of estragole (73.0%) and the presence of methyl eugenol (7.2%) in basil essential oil are particularly relevant from a safety perspective when considering potential cosmetic or dermatological applications. The toxicological significance of these phenylpropanoids depends on exposure conditions, including dose, duration, and route of administration [7,30]. Importantly, safety cannot be inferred from the cell-free bioactivity assays performed in the present study; therefore, the present findings should not be interpreted as demonstrating the safety of EOB for topical use. Any future formulation containing this essential oil would require exposure-specific toxicological assessment and evaluation of compliance with applicable regulatory requirements.

### 2.2. Cyclooxygenase-2 (COX-2) Inhibitory Activity of Basil and Clove Essential Oils

**Figure 3:**
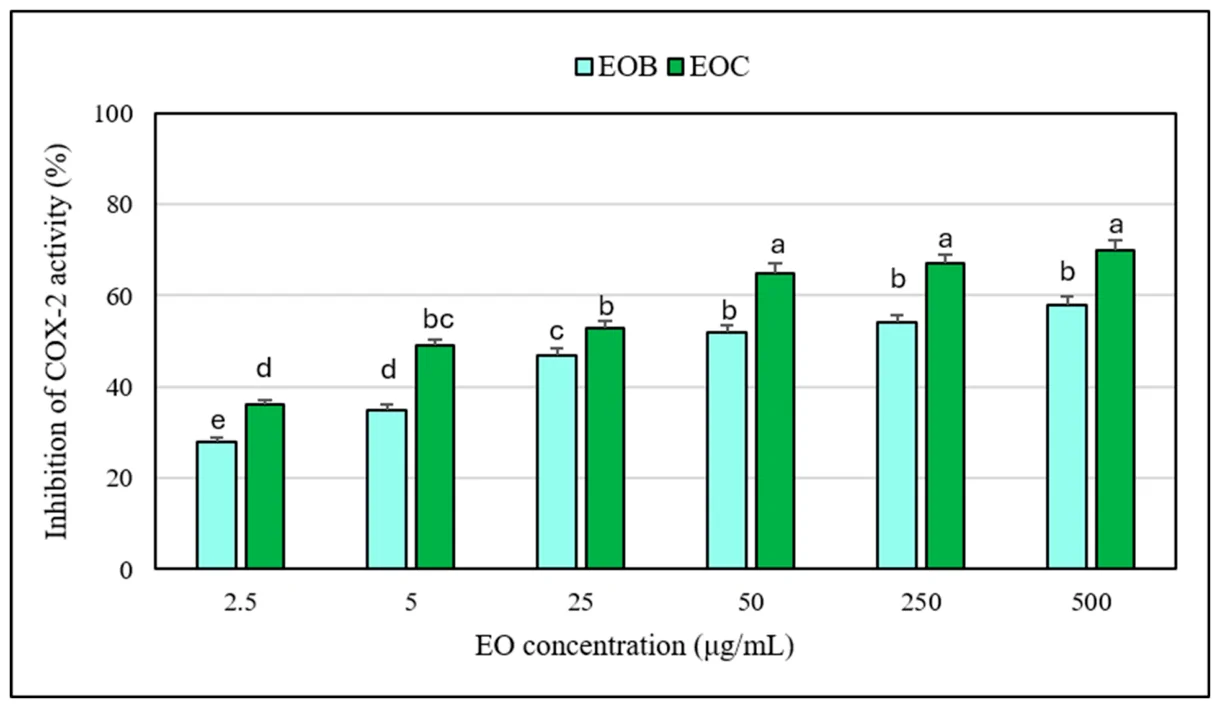
Cyclooxygenase-2 (COX-2) inhibitory activity of individual basil and clove essential oils. The inhibitory effects of essential oils obtained from the aerial parts of *Ocimum basilicum* L. (EOB) and the floral buds of *Syzygium aromaticum* L. (EOC) were evaluated at different concentrations ranging from 2.5 to 500 µg/mL. Results are expressed as COX-2 inhibition (%), relative to the untreated enzyme control (100% enzyme activity) and presented as mean ± standard deviation (SD). Different letters above the bars indicate statistically significant differences among treatments, according to Tukey’s multiple comparison test (*p* < 0.05).

The inhibitory potential of basil essential oil (EOB) and clove essential oil (EOC) against cyclooxygenase-2 (COX-2) activity was evaluated over a concentration range of 2.5–500 µg/mL (Figure 3). The enzyme activity measured in the absence of essential oils was defined as 100%, and the inhibitory effect was expressed as the percentage inhibition of COX-2 activity. Both essential oils inhibited COX-2 activity in a concentration-dependent manner; however, inhibitory activity differed substantially between the tested oils.

EOB demonstrated a moderate inhibitory effect, with COX-2 inhibition increasing progressively from 28% at 2.5 µg/mL to 58% at 500 µg/mL. The calculated half-maximal inhibitory concentration (IC_50_) of EOB was 40.3 ± 0.8 µg/mL. In contrast, EOC exhibited markedly stronger inhibitory activity, with COX-2 inhibition increasing from 36% at the lowest concentration tested to 70% at 500 µg/mL. The IC_50_ value determined for EOC (9.0 ± 0.5 µg/mL) was approximately 4.5-fold lower than that of EOB, confirming its substantially higher inhibitory activity toward COX-2. Celecoxib, the reference COX-2 inhibitor, was also evaluated over a concentration range of 0–30 µg/mL and exhibited an IC_50_ of 2.4 ± 0.1 µg/mL. Based on IC_50_ values obtained under the same experimental conditions, celecoxib exhibited approximately 3.8-fold greater inhibitory potency than EOC and approximately 16.8-fold greater inhibitory potency than EOB. A separately prepared celecoxib solution at 25 µg/mL served as the positive control and produced 92% COX-2 inhibition.

Previous studies have also reported COX-2 inhibitory activity of plant essential oils, although direct quantitative comparisons are limited by differences in assay design and the units used to express essential oil concentrations. Fokou et al. [31] reported a COX-2 IC_50_ of 0.22 µL/mL for *Ocimum basilicum* essential oil, while *O. gratissimum* essential oil exhibited a COX-2 IC_50_ of 0.27 µL/mL. Because these values were expressed on a volume basis and obtained using a colorimetric assay with ovine COX-2, direct quantitative comparison with the µg/mL values from the present study, obtained using a fluorometric assay with human recombinant COX-2, is not appropriate. Li et al. [32] reported an IC_50_ of 30.9 µg/mL for the inhibition of COX-2 activity by *Houttuynia cordata* essential oil. This value is of the same order of magnitude as that obtained for EOB in the present study (40.3 ± 0.8 µg/mL), whereas EOC exhibited a substantially lower IC_50_ (9.0 ± 0.5 µg/mL). However, Li et al. evaluated COX-2 activity in LPS-induced mouse peritoneal macrophages rather than in a cell-free recombinant enzyme system. Accordingly, these comparisons should be interpreted cautiously because differences in essential oil composition, enzyme source, biological model, substrate, and assay format may markedly influence apparent inhibitory potency.

Differences in COX-2-inhibitory potency between EOC and EOB may reflect their distinct overall phytochemical profiles [33]. Published studies have associated eugenol and other essential-oil constituents with modulation of inflammation-related pathways and enzymes involved in arachidonic acid metabolism [34,35]. However, the activities of the individual constituents of EOB and EOC were not examined in the present study. Consequently, the observed differences in COX-2 inhibition cannot be assigned specifically to eugenol, estragole, (E)-β-caryophyllene, or any other individual compound and should instead be interpreted at the level of the chemically complex essential oils. COX-2 is also relevant to skin-aging processes because UV-induced oxidative stress and inflammatory signalling can increase COX-2 activity and prostaglandin production, contributing to inflammation-associated tissue damage and extracellular matrix remodelling [36,37,38,39].

**Figure 4:**
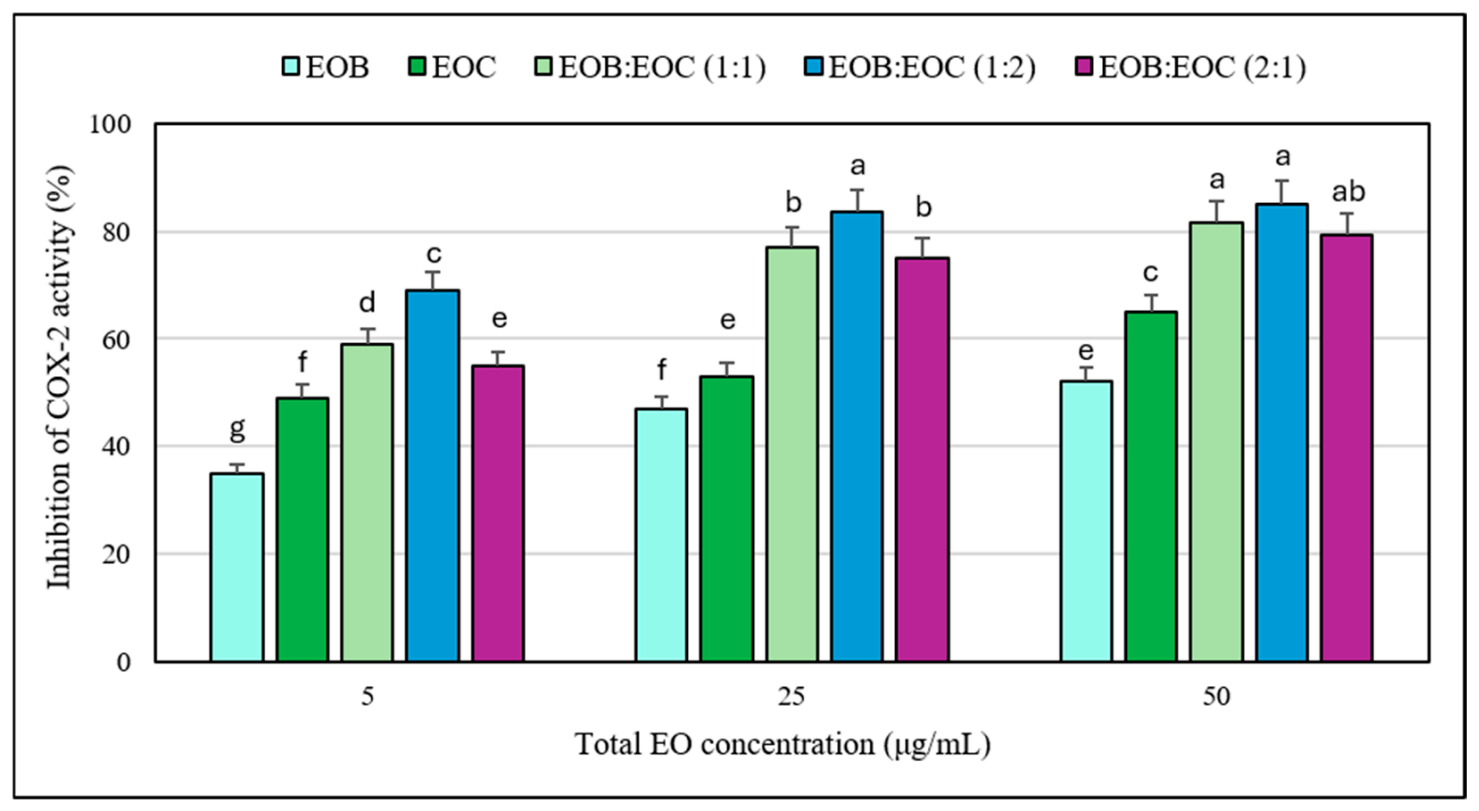
Cyclooxygenase-2 (COX-2) inhibitory activity of basil (EOB) and clove (EOC) essential oils and their binary mixtures. EOB and EOC were tested individually at concentrations of 5, 25, and 50 µg/mL, while binary EOB:EOC mixtures (1:1, 1:2, and 2:1, *v*/*v*) were evaluated at the same total essential oil concentrations. The previously presented individual EOB and EOC data at 5, 25, and 50 µg/mL are replotted here to facilitate direct comparison with the binary mixtures. Results are expressed as COX-2 inhibition (%) and presented as mean ± SD from three independent experiments, each with two technical replicates. The effects of formulation and concentration were evaluated by ANOVA followed by Tukey’s multiple comparison test. Different letters above the bars indicate significant differences among all formulation–concentration combinations (*p* < 0.05).

To assess possible interactions between the essential oils, binary mixtures of EOB and EOC were evaluated at total essential oil concentrations of 5, 25, and 50 µg/mL using three volumetric ratios (1:1, 1:2, and 2:1, *v*/*v*) (Figure 4). For direct comparison with the binary mixtures, the previously presented individual EOB and EOC data at the corresponding concentrations were replotted in Figure 4. At 5 µg/mL, COX-2 inhibition by the binary mixtures ranged from 54.9% to 69.0%, with the highest activity observed for the 1:2 EOB:EOC mixture. At 25 µg/mL, inhibition ranged from 75.0% to 83.5%, whereas at 50 µg/mL the mixtures produced 79.3–85.1% inhibition. At each total essential oil concentration, the 1:2 EOB:EOC mixture exhibited the highest inhibitory activity among the three tested ratios.

**Figure 5:**
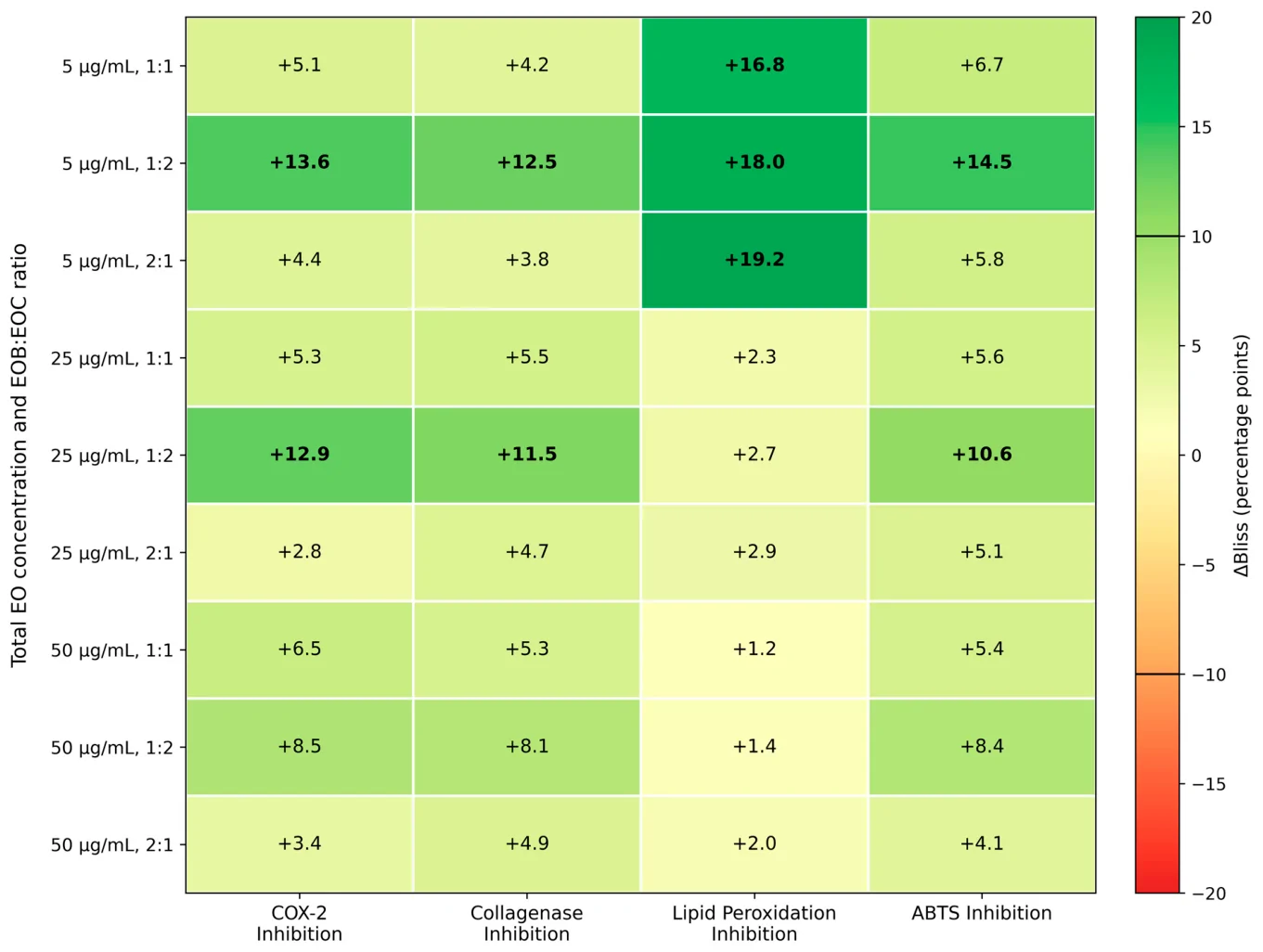
Interaction effects of basil (EOB) and clove (EOC) essential oils, based on Bliss independence analysis. The heatmap summarizes excess over Bliss (ΔBliss) values for four tested bioactivities: cyclooxygenase-2 (COX-2) inhibition, collagenase inhibition, lipid peroxidation inhibition, and ABTS^•+^ radical scavenging activity. Binary mixtures were evaluated at EOB:EOC volumetric ratios (*v*/*v*) of 1:1, 1:2, and 2:1 and at total essential oil concentrations of 5, 25, and 50 µg/mL. ΔBliss was calculated as the difference between the experimentally observed mixture response and the response expected under the Bliss independence model. ΔBliss values > +10 percentage points were considered consistent with synergistic interactions; values from −10 to +10 percentage points, inclusive, were considered to indicate no substantial deviation from Bliss independence; and values < −10 percentage points were considered consistent with antagonistic interactions. Values exceeding the predefined synergy threshold of +10 percentage points are shown in bold.

Interactions between EOB and EOC were further evaluated using the Bliss independence model (Figure 5; Table S1). All ΔBliss values were positive, indicating that the experimentally observed COX-2 inhibitory effects of all tested binary mixtures exceeded the corresponding effects expected under Bliss independence. At 5 µg/mL, ΔBliss values were +5.1, +13.6, and +4.4 percentage points for the 1:1, 1:2, and 2:1 mixtures, respectively. At 25 µg/mL, the corresponding values were +5.3, +12.9, and +2.8 percentage points, while at 50 µg/mL they were +6.5, +8.5, and +3.4 percentage points. However, only the 1:2 EOB:EOC mixtures at 5 and 25 µg/mL exceeded the predefined +10-percentage-point threshold and were therefore considered consistent with synergistic interactions according to the operational criterion adopted in this study. The remaining combinations showed positive ΔBliss values within the −10 to +10 percentage-point interval and were interpreted as showing no substantial deviation from Bliss independence. No antagonistic interactions were observed under the tested conditions.

In particular, the 1:2 formulation, containing the higher proportion of EOC, produced the largest positive deviation from the Bliss-independence expectation at the lower and intermediate concentrations. This pattern is consistent with the substantially greater COX-2 inhibitory potency of EOC when tested individually, as reflected by its lower IC_50_ value compared with EOB. Nevertheless, the positive ΔBliss values cannot be attributed solely to the greater intrinsic activity of EOC, because the Bliss model compares the experimentally observed mixture response with the response expected from the individual effects of both oils at the component concentrations present in the respective mixture.

Because the activities of isolated constituents and their specific molecular targets were not examined, the positive Bliss deviations cannot be attributed to individual compounds or defined molecular mechanisms and should therefore be interpreted as quantitative evidence of interaction at the assay-response level.

The absence of ΔBliss values exceeding +10 percentage points at 50 µg/mL, despite the relatively high absolute COX-2 inhibition produced by the mixtures, may partly reflect the approach of the concentration–response relationships toward higher inhibitory levels, thereby restricting the remaining response range available for detecting further enhancement. Accordingly, high absolute inhibitory activity should not itself be interpreted as evidence of synergism; within the Bliss framework applied here, synergistic interaction was defined descriptively by a sufficiently large positive deviation from the response expected under independent action. Overall, EOB–EOC interactions in the COX-2 assay were concentration- and ratio-dependent rather than uniformly synergistic. These findings emphasize the importance of evaluating essential oil combinations across multiple concentrations and mixture ratios rather than relying on a single high-concentration endpoint.

### 2.3. Inhibition of Collagenase Activity by the Investigated Essential Oils

**Figure 6:**
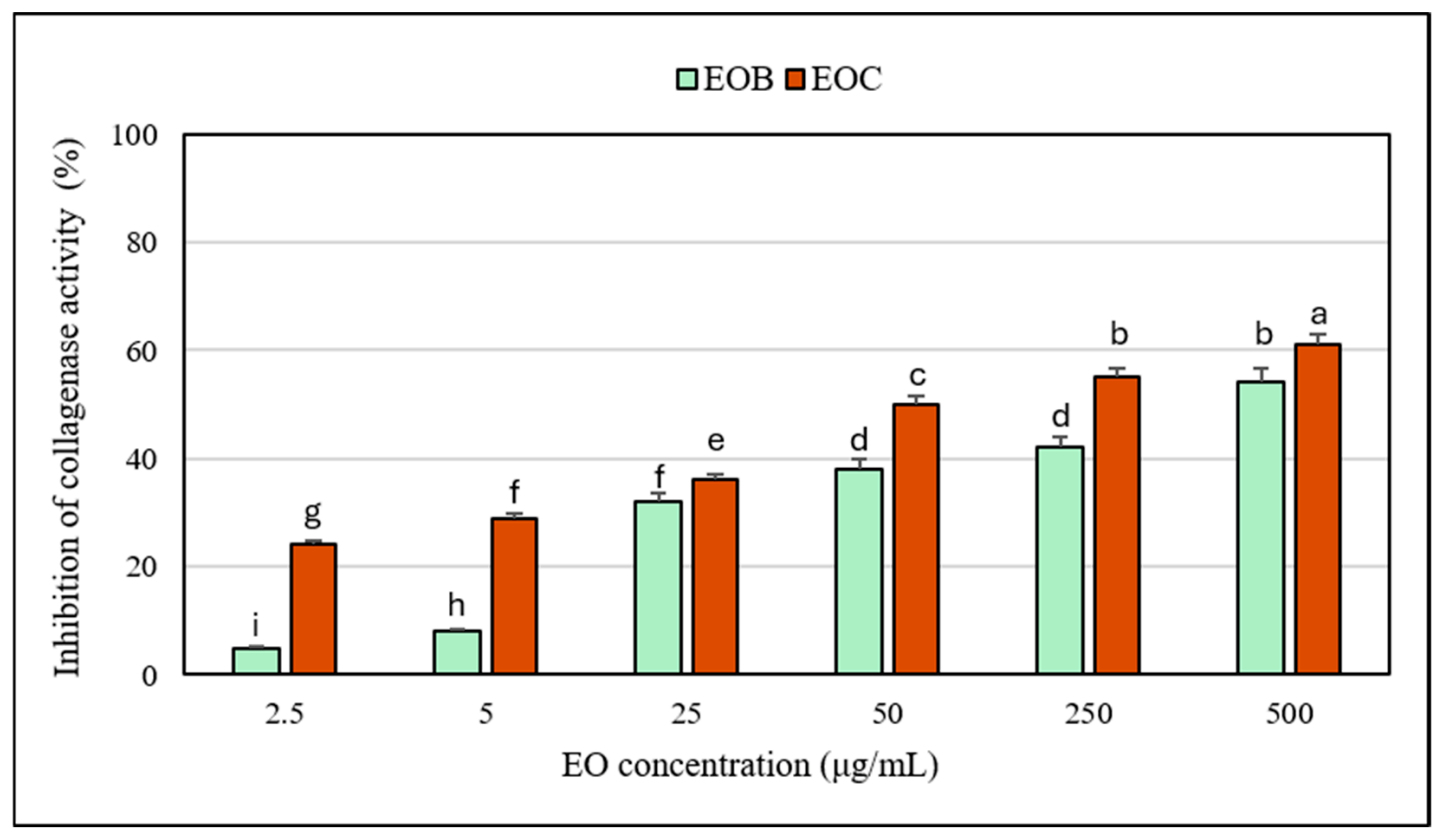
Collagenase inhibitory activity of individual basil and clove essential oils. The inhibitory effects of essential oils obtained from the aerial parts of *Ocimum basilicum* L. (EOB) and the floral buds of *Syzygium aromaticum* L. (EOC) were evaluated at different concentrations ranging from 2.5 to 500 µg/mL. Results are expressed as collagenase inhibition (%), relative to the untreated enzyme control (100% enzyme activity) and presented as mean ± standard deviation (SD). Different letters above the bars indicate statistically significant differences among treatments, according to Tukey’s multiple comparison test (*p* < 0.05).

The collagenase inhibitory activity of basil and clove EOs was evaluated over a concentration range of 2.5–500 µg/mL (Figure 6). Values were expressed as collagenase inhibition (%) relative to the untreated enzyme control, which represents 100% collagenase activity. Both essential oils showed concentration-dependent inhibition; however, their inhibitory potential differed substantially. Basil essential oil exhibited a gradual increase in collagenase inhibition with increasing concentration, ranging from 5% at 2.5 µg/mL to 54% at 500 µg/mL. Although the inhibitory response increased consistently across the tested concentration range, EOB displayed relatively moderate collagenase inhibitory activity. In contrast, clove essential oil demonstrated markedly stronger activity, with inhibition values increasing from 24% at 2.5 µg/mL to 61% at 500 µg/mL. EOC showed higher inhibitory effects at all tested concentrations, indicating greater collagenase-inhibitory potential than basil essential oil. The difference in inhibitory activity between the oils was further confirmed by IC_50_ determination. The IC_50_ value for EOB was 348 ± 10.5 µg/mL, whereas EOC exhibited a substantially lower IC_50_ value of 50.5 ± 0.8 µg/mL. Thus, EOC exhibited an approximately 6.9-fold lower IC_50_ than EOB, confirming its greater collagenase-inhibitory potency under the experimental conditions applied.

1,10-Phenanthroline, used as the reference collagenase inhibitor, was also evaluated over a concentration range of 0–200 µg/mL and showed an IC_50_ of 30.2 ± 0.8 µg/mL. Based on IC_50_ values obtained under the same experimental conditions, 1,10-phenanthroline showed approximately 1.7-fold greater inhibitory potency than EOC and approximately 11.5-fold greater inhibitory potency than EOB. At the positive-control concentration of 0.8 mM (equivalent to approximately 144.2 µg/mL), 1,10-phenanthroline produced 94% inhibition of collagenase activity, confirming adequate assay performance under the applied experimental conditions.

Previous studies have likewise demonstrated substantial variability in collagenase inhibition among plant-derived preparations. Baković et al. [40] reported collagenase inhibition ranging from less than 20% to 72.42% for ethanolic extracts of different *Ocimum* cultivars tested at 3.33 mg/mL, with Purple basil showing the highest inhibitory activity. Because these authors investigated ethanolic extracts rather than essential oils and reported inhibition at a single concentration rather than IC_50_ values, direct quantitative comparison with the present EOB results is limited. Nevertheless, their findings demonstrate marked cultivar-dependent variation in the collagenase-inhibitory activity of *Ocimum* preparations. Quantitative data reported for other essential oils provide additional context for the IC_50_ values obtained in the present study. Fraternale et al. [41] reported a collagenase IC_50_ of 36.99 ± 1.52 µg/mL for *Helichrysum italicum* essential oil. Essential oils obtained from different organs of *Syzygium cumini* were reported to inhibit collagenase with IC_50_ values of 20.80 µg/mL for seed oil, 29.39 µg/mL for leaf oil, 67.96 µg/mL for bark oil, and 173.90 µg/mL for fruit oil [42]. The IC_50_ obtained for EOC in the present study (50.5 ± 0.8 µg/mL) was therefore within the range reported for these collagenase-inhibitory essential oils and was of the same order of magnitude as that reported for *H. italicum* essential oil. In contrast, EOB exhibited markedly weaker collagenase-inhibitory potency, as reflected by its substantially higher IC_50_ value. Nevertheless, direct comparisons should be interpreted cautiously because differences in enzyme source, substrate, assay format, and essential oil composition may substantially affect the measured IC_50_ values.

The IC_50_ value obtained for the reference inhibitor 1,10-phenanthroline (30.2 ± 0.8 µg/mL) was also consistent with values reported in previous collagenase inhibition studies. Hartmann et al. reported an IC_50_ of 42.86 ± 1.35 µg/mL (238.1 ± 3.4 µM) for 1,10-phenanthroline, whereas Nitulescu et al. reported an IC_50_ of 114.810 µM (approximately 20.7 µg/mL) [43,44]. The IC_50_ determined in the present study was therefore intermediate between these previously reported values. Differences among studies may reflect variations in enzyme and substrate concentrations, incubation conditions, reaction time, and other assay-specific parameters.

Differences in collagenase-inhibitory potency between EOC and EOB may reflect their distinct overall phytochemical profiles. Published studies have associated eugenol and other phenolic compounds with antioxidant and enzyme-modulating activities and have proposed interactions with enzyme structures through hydrogen bonding and hydrophobic interactions as possible mechanisms of collagenase inhibition [16,45,46]. However, the contribution of eugenol or any other individual constituent to the activity observed in the present study cannot be established from the current experimental design. Likewise, although EOB represented an estragole-dominant chemotype, the lower collagenase-inhibitory potency of this oil cannot be attributed specifically to estragole or to the abundance of any individual constituent. The observed differences should therefore be interpreted at the level of the chemically complex essential oils, while constituent-level explanations remain hypothetical.

**Figure 7:**
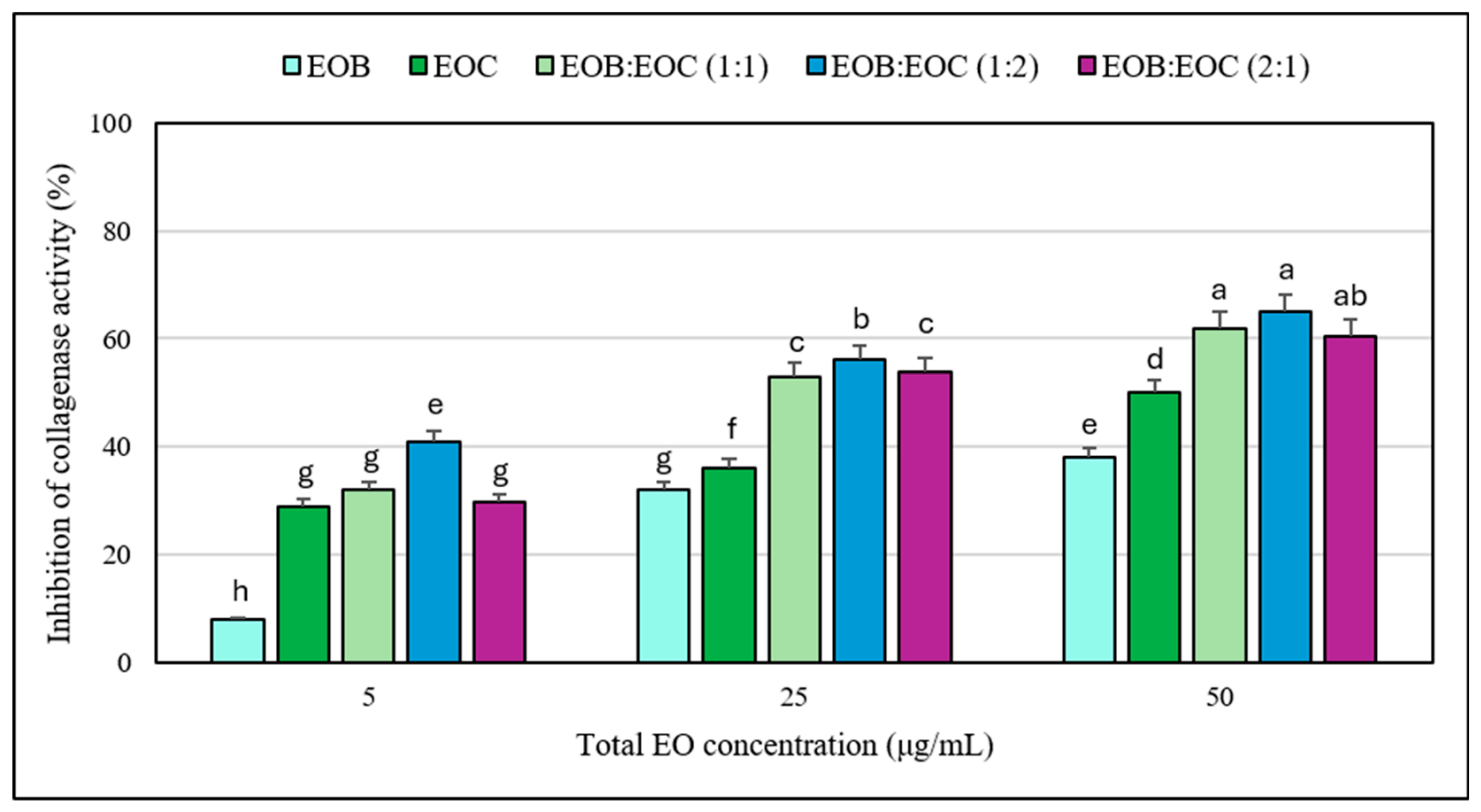
Collagenase inhibitory activity of basil (EOB) and clove (EOC) essential oils and their binary mixtures. EOB and EOC were tested individually at concentrations of 5, 25, and 50 µg/mL, while binary EOB:EOC mixtures (1:1, 1:2, and 2:1, *v*/*v*) were evaluated at the same total essential oil concentrations. The previously presented individual EOB and EOC data at 5, 25, and 50 µg/mL are replotted here to facilitate direct comparison with the binary mixtures. Results are expressed as collagenase inhibition (%) and presented as mean ± SD from three independent experiments, each with two technical replicates. The effects of formulation and concentration were evaluated by ANOVA followed by Tukey’s multiple comparison test. Different letters above the bars indicate significant differences among all formulation–concentration combinations (*p* < 0.05).

To test whether combining both essential oils could enhance collagenase inhibition, we evaluated binary mixtures of EOB and EOC at total essential oil concentrations of 5, 25, and 50 µg/mL using three volumetric ratios (1:1, 1:2, and 2:1, *v*/*v*) (Figure 7). For direct comparison with the binary mixtures, the previously presented individual EOB and EOC data at the corresponding concentrations were replotted in Figure 7. At 5 µg/mL, collagenase inhibition by the mixtures ranged from 29.8% to 41.0%, with the highest activity observed for the 1:2 EOB:EOC mixture. At 25 µg/mL, inhibition ranged from 53.0% to 56.0%, whereas at 50 µg/mL the mixtures produced 60.5–65.0% inhibition. At each total essential oil concentration, the 1:2 EOB:EOC mixture exhibited the highest collagenase inhibitory activity among the three tested ratios.

Interactions between EOB and EOC were further evaluated using the Bliss independence model (Figure 5; Table S2). All ΔBliss values were positive, indicating that the experimentally observed collagenase inhibitory effects of all tested binary mixtures exceeded the corresponding effects expected under Bliss independence. At 5 µg/mL, ΔBliss values were +4.2, +12.5, and +3.8 percentage points for the 1:1, 1:2, and 2:1 mixtures, respectively. At 25 µg/mL, the corresponding values were +5.5, +11.5, and +4.7 percentage points, whereas at 50 µg/mL they were +5.3, +8.1, and +4.9 percentage points. However, only the 1:2 EOB:EOC mixtures at 5 and 25 µg/mL exceeded the predefined +10-percentage-point threshold and were therefore considered consistent with synergistic interactions according to the operational criterion adopted in this study. The remaining combinations showed positive ΔBliss values within the −10 to +10 percentage-point interval and were interpreted as showing no substantial deviation from Bliss independence. No antagonistic interactions were observed under the tested conditions. The concentration- and ratio-dependent interaction pattern indicates that strong absolute collagenase inhibition and synergistic interaction should be considered distinct characteristics of the mixture response. Although EOC exhibited substantially greater collagenase inhibitory potency when tested individually, the 1:2 EOB:EOC mixtures at 5 and 25 µg/mL exceeded the responses expected from the individual effects of both oils under the Bliss independence model. Because the activities of isolated constituents and their specific molecular targets were not examined, the positive Bliss deviations should be interpreted as quantitative evidence of interaction at the assay-response level rather than as proof of a specific molecular mechanism.

From a dermatological perspective, collagenase inhibition is relevant to processes associated with extracellular matrix degradation and skin aging. However, the present findings were obtained in a cell-free enzyme assay and should not be interpreted as demonstrating protection of the extracellular matrix or anti-aging efficacy in biological tissues. The observed activity therefore supports further investigation of basil–clove essential oil mixtures in cellular and skin-relevant models, together with appropriate evaluation of their efficacy and safety [6,47].

To the best of our knowledge, the interaction between basil (*Ocimum basilicum* L.) and clove (*Syzygium aromaticum* L.) essential oils in the collagenase assay has not been systematically characterized across multiple mixture ratios and concentrations using Bliss independence analysis. Previous investigations of these essential oils have primarily focused on antioxidant, antimicrobial, and anti-inflammatory properties, whereas their effects on collagenase remain comparatively less explored.

### 2.4. Antioxidant Activity of the Tested Essential Oils

#### 2.4.1. Inhibition of Linoleic Acid Peroxidation

**Figure 8:**
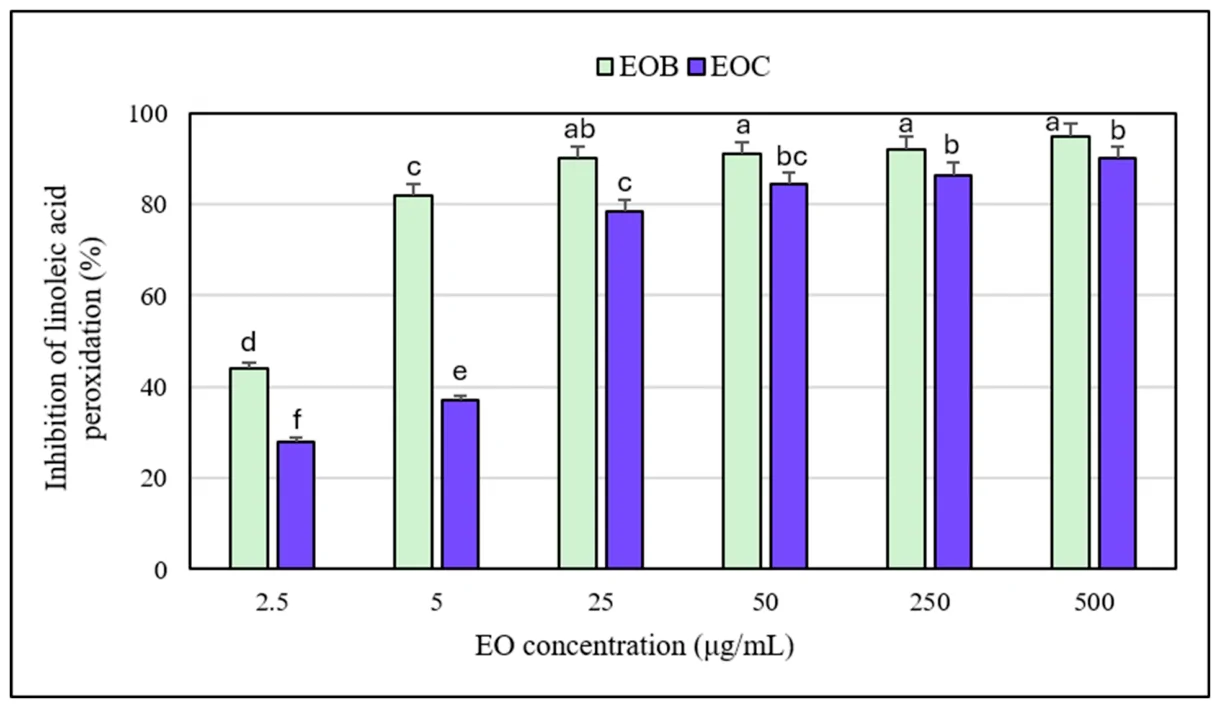
Inhibitory activity of individual basil and clove essential oils against linoleic acid peroxidation. The essential oils obtained from the aerial parts of *Ocimum basilicum* L. (EOB) and the floral buds of *Syzygium aromaticum* L. (EOC) were evaluated at concentrations ranging from 2.5 to 500 µg/mL. Results are expressed as inhibition of linoleic acid peroxidation (%) and presented as mean ± standard deviation (SD). Different letters above the bars indicate statistically significant differences among treatments, according to Tukey’s multiple comparison test (*p* < 0.05).

The inhibitory activity of basil (*Ocimum basilicum* L.; EOB) and clove (*Syzygium aromaticum* L.; EOC) essential oils against linoleic acid peroxidation increased in a concentration-dependent manner throughout the tested concentration range (2.5–500 µg/mL) (Figure 8). Both essential oils exhibited pronounced antioxidant activity; however, EOB consistently demonstrated higher inhibitory efficiency than EOC at all tested concentrations, indicating a greater capacity to prevent lipid oxidation in this assay. At the lowest concentration evaluated (2.5 µg/mL), EOB inhibited linoleic acid peroxidation by 44%, whereas EOC showed 28% inhibition. As concentration increased, both oils showed progressively greater inhibition of linoleic acid peroxidation. At the highest concentration (500 µg/mL), inhibition reached 95% for EOB and 90% for EOC, confirming the strong lipid peroxidation-inhibitory activity of both essential oils. Nevertheless, the consistently higher inhibition values obtained for EOB across the entire concentration range indicate superior effectiveness of basil essential oil in this lipid oxidation model.

The calculated IC_50_ values further confirmed the difference in antioxidant capacity between the oils. EOB exhibited an IC_50_ value of 2.9 ± 0.2 µg/mL, whereas EOC showed an IC_50_ value of 11.3 ± 0.4 µg/mL. BHT, used as the reference antioxidant, was additionally evaluated over the concentration range of 0–100 µg/mL and showed an IC_50_ value of 6.3 ± 0.2 µg/mL. Thus, based on IC_50_ values obtained under the same experimental conditions, the inhibitory potency followed the order EOB > BHT > EOC. EOB exhibited an approximately 2.2-fold lower IC_50_ than BHT, whereas EOC showed an approximately 1.8-fold higher IC_50_ than BHT. At 20 µg/mL, BHT, included as the positive control point in the assay, produced 80% inhibition of linoleic acid peroxidation.

Hussain et al. [48] reported that *O. basilicum* essential oils obtained in different seasons inhibited linoleic acid oxidation by 80.3–91.2%. The winter and spring oils produced 91.2% and 90.3% inhibition, respectively, values comparable to the 91.1% inhibition observed for BHT in their experimental system. Their basil essential oils were predominantly linalool-rich (56.7–60.6%), in contrast to the estragole-dominant EOB investigated in the present study, further illustrating the influence of chemotype on antioxidant behaviour. For clove essential oil, Gülçin et al. [49] reported 97.3% inhibition of lipid peroxidation in a linoleic acid emulsion at 15 µg/mL, whereas BHT produced 99.7% inhibition at 45 µg/mL. These results confirm the strong lipid-phase antioxidant activity previously reported for the clove oil. However, the absolute values should not be directly compared with those obtained in the present study because the experimental protocols differed in oxidation initiation, incubation conditions, and analytical readout. In the present hemoglobin-induced linoleic acid peroxidation assay, EOC exhibited an IC_50_ of 11.3 ± 0.4 µg/mL, whereas EOB showed a lower IC_50_ of 2.9 ± 0.2 µg/mL. These differences across studies therefore reinforce that antioxidant potency in lipid systems depends strongly on both essential oil composition and the specific experimental model used.

Lipid peroxidation is a fundamental oxidative process involving the formation and propagation of lipid-derived free radicals, leading to membrane damage, disruption of cellular homeostasis, and amplification of oxidative stress-associated signalling pathways. In skin tissues, excessive lipid oxidation contributes to impaired barrier function, inflammatory activation, and progression of photoaging-related alterations. Consequently, inhibiting lipid peroxidation is an important biological property of natural compounds considered for skin-protective and anti-aging applications [6,36].

The higher activity of EOB observed in the present study suggests that antioxidant efficiency in lipid systems is determined not only by radical scavenging capacity but also by physicochemical properties affecting antioxidant localization, mobility, and interaction with lipid substrates. Although clove essential oil is characterized by a high content of eugenol, a phenolic compound with well-established antioxidant activity, the effectiveness of antioxidants in lipid oxidation models depends on additional factors, including lipophilicity, partitioning behaviour, molecular diffusion, and accessibility to lipid-derived radicals [16,26,50]. Eugenol present in clove essential oil possesses a hydroxylated aromatic structure enabling hydrogen donation and stabilization of phenoxy radicals through resonance delocalization. These properties explain its strong antioxidant activity in several chemical and biological models. However, in lipid-based systems, antioxidant potential may also depend on the ability of molecules to reach the site of radical generation and efficiently interfere with propagation reactions. Therefore, a compound exhibiting high radical scavenging activity does not necessarily demonstrate superior inhibition of lipid oxidation compared with chemically diverse mixtures containing moderately active constituents with more favourable distribution properties [50]. The greater activity of EOB in the present assay may reflect the combined contribution of its volatile constituents and their physicochemical behaviour within the linoleic acid oxidation system. However, the activities of individual EOB constituents were not examined, and the higher lipid peroxidation-inhibitory potency of EOB cannot therefore be assigned to specific compounds. Basil essential oils exhibit considerable chemical variability depending on cultivar, geographical origin, and environmental conditions; however, their biological activity is frequently associated with the presence of linalool, methyl chavicol (estragole), eugenol derivatives, and other oxygenated terpenoid compounds capable of contributing to antioxidant responses [25,35]. The differences observed between EOB and EOC highlight an important aspect of essential oil research: antioxidant activity is a context-dependent property influenced by both chemical composition and the analytical model used. Electron transfer-based assays primarily reflect radical neutralizing capacity, whereas lipid peroxidation models also incorporate processes associated with molecular transport, phase distribution, and interaction with lipid substrates. Consequently, the superior performance of basil essential oil in the present assay does not contradict the well-documented antioxidant activity of eugenol-rich clove oil but demonstrates that different essential oils may exhibit distinct strengths depending on the oxidative environment tested [10,50].

**Figure 9:**
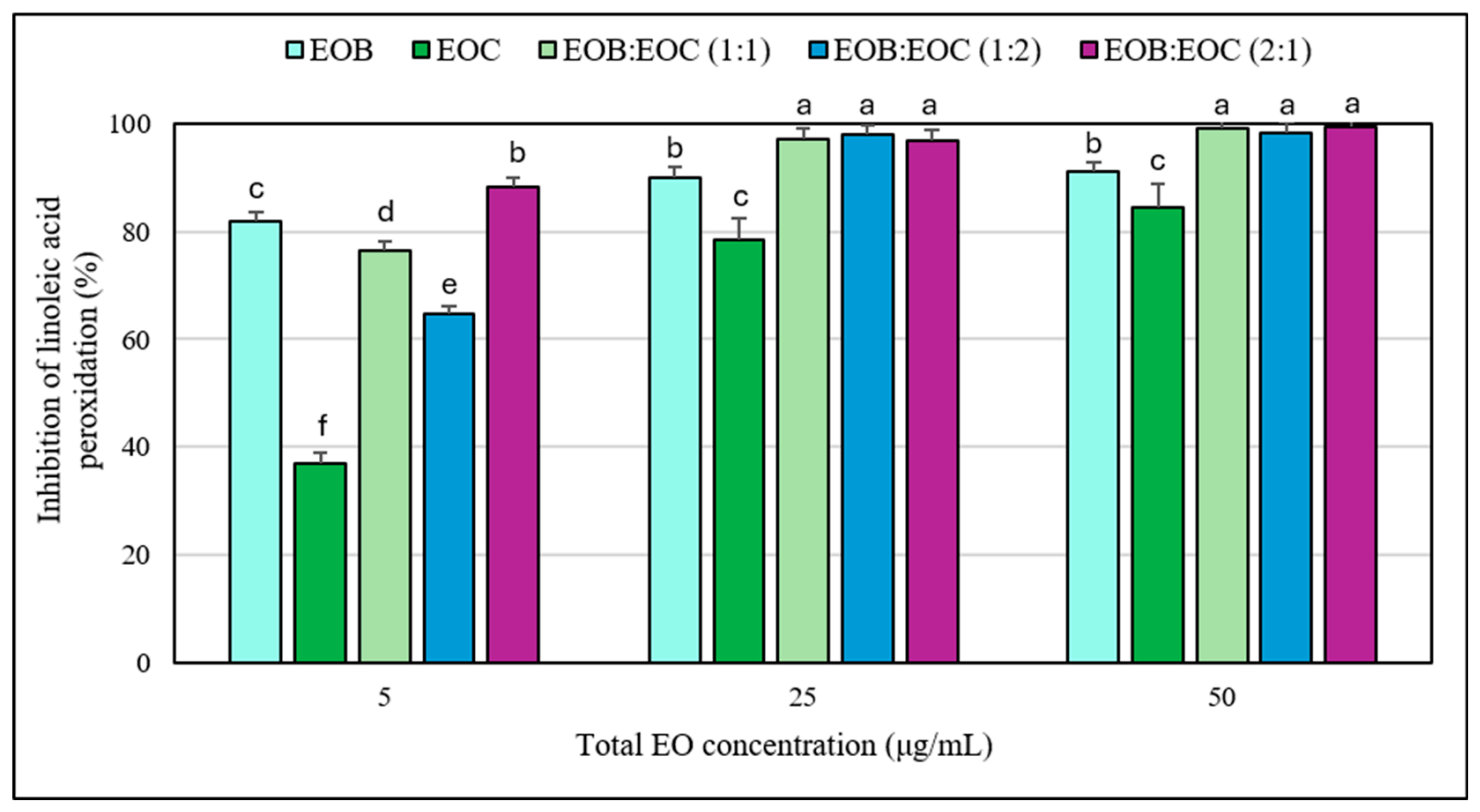
Inhibitory effects of basil (EOB) and clove (EOC) essential oils and their binary mixtures on linoleic acid peroxidation. EOB and EOC were tested individually at concentrations of 5, 25, and 50 µg/mL, while binary EOB:EOC mixtures (1:1, 1:2, and 2:1, *v*/*v*) were evaluated at the same total essential oil concentrations. The previously presented individual EOB and EOC data at 5, 25, and 50 µg/mL are replotted here to facilitate direct comparison with the binary mixtures. Results are expressed as linoleic acid peroxidation inhibition (%) and presented as mean ± SD from three independent experiments, each with two technical replicates. The effects of formulation and concentration were evaluated by ANOVA followed by Tukey’s multiple comparison test. Different letters above the bars indicate significant differences among all formulation–concentration combinations (*p* < 0.05).

To test whether combining both essential oils could improve antioxidant activity, binary mixtures of EOB and EOC were evaluated at total essential oil concentrations of 5, 25, and 50 µg/mL using three volumetric ratios (1:1, 1:2, and 2:1, *v*/*v*) (Figure 9). For direct comparison with the binary mixtures, the previously presented individual EOB and EOC data at the corresponding concentrations were replotted in Figure 9. At 5 µg/mL, inhibition of linoleic acid peroxidation by the mixtures ranged from 64.7% to 88.1%, with the highest activity observed for the 2:1 EOB:EOC mixture. At 25 µg/mL, inhibition ranged from 96.9% to 97.8%, whereas at 50 µg/mL the mixtures produced 98.2–99.4% inhibition. The predominance of EOB in the most active mixture at 5 µg/mL is consistent with the greater lipid peroxidation-inhibitory activity of basil essential oil observed when the oils were tested individually.

Interactions between EOB and EOC were further evaluated using the Bliss independence model (Figure 5; Table S3). All ΔBliss values were positive, indicating that the experimentally observed inhibitory effects of all tested binary mixtures exceeded the corresponding effects expected under Bliss independence. At 5 µg/mL, ΔBliss values were +16.8, +18.0, and +19.2 percentage points for the 1:1, 1:2, and 2:1 mixtures, respectively. Thus, all three mixtures at this concentration exceeded the predefined +10-percentage-point threshold and were considered consistent with synergistic interactions according to the operational criterion adopted in this study. At 25 µg/mL, ΔBliss values decreased markedly to +2.3, +2.7, and +2.9 percentage points for the 1:1, 1:2, and 2:1 mixtures, respectively, while at 50 µg/mL the corresponding values were +1.2, +1.4, and +2.0 percentage points. These combinations therefore showed no substantial deviation from Bliss independence. No antagonistic interactions were observed under the tested conditions. The concentration-dependent pattern was particularly pronounced in the lipid peroxidation assay. At 5 µg/mL, all three mixture ratios showed substantial positive deviations from the Bliss-independence expectation, with the strongest response observed for the 2:1 formulation. In contrast, at 25 and 50 µg/mL, the observed mixture effects approached 100% inhibition, while the corresponding Bliss-expected effects were already very high (94.0–95.1% and 96.8–97.9%, respectively). Consequently, the remaining response range available for further enhancement was highly restricted. Similar concentration-dependent behavior has been described for complex phytochemical mixtures, in which high baseline activity and response saturation may reduce the measurable magnitude of interaction effects [51,52]. Accordingly, the small positive ΔBliss values obtained at 25 and 50 µg/mL should not be interpreted as evidence of reduced absolute antioxidant activity; rather, they indicate that the observed responses were close to those predicted under Bliss independence. The strong positive deviations at 5 µg/mL may reflect complementary contributions from chemically diverse constituents in the two essential oils. Both EOB and EOC contain compounds that may interrupt oxidative chain reactions through hydrogen donation, radical stabilization, and interactions within lipid environments. Eugenol-rich clove essential oil provides strong phenolic antioxidant activity, whereas EOB contains phenylpropanoid and terpenoid constituents that may contribute through physicochemical properties affecting localization and interaction with lipid-derived radicals. However, coexistence of compounds acting through partially overlapping mechanisms may also explain why the magnitude of the Bliss deviation decreased markedly once the overall antioxidant response approached saturation [50,51]. These interpretations remain mechanistic hypotheses because the present study did not examine the activities of isolated constituents or their specific interactions within the lipid oxidation system.

Regarding formulation design, the observed interaction pattern indicates that the most effective EOB:EOC ratio among those tested depended on both concentration and the biological endpoint under investigation. At the lowest total concentration, the 2:1 mixture, containing a higher proportion of EOB, produced both the greatest absolute inhibition and the largest ΔBliss value. This finding is consistent with EOB’s superior performance in the individual lipid peroxidation assay. At higher concentrations, however, all three formulations produced similarly high absolute inhibition, and their ΔBliss values remained within the range defined as no substantial deviation from Bliss independence. Such findings reinforce the need to evaluate essential oil combinations across multiple concentrations rather than infer interaction behavior from a single high-concentration condition [23,53,54]. From a dermatological perspective, inhibiting lipid peroxidation is an important protective mechanism against oxidative damage associated with skin aging and environmental stress. Reactive oxygen species generated during ultraviolet exposure and chronic oxidative stress may promote membrane lipid oxidation, impair epidermal barrier function, and activate inflammatory pathways involved in photoaging progression. Therefore, essential oils with lipid-protective activity may warrant further investigation in skin-protective formulations, provided their efficacy and safety are confirmed in more biologically relevant models [6,36].

The present results demonstrate that both basil and clove essential oils exhibit strong inhibitory activity against linoleic acid peroxidation, with basil essential oil showing greater potency, as reflected by its lower IC_50_ value and higher inhibition across the individual-oil concentration range. Clove essential oil remained an active antioxidant system, consistent with its eugenol-rich phytochemical profile and previously documented radical scavenging properties [16,26]. Importantly, all tested EOB:EOC combinations produced positive ΔBliss values, whereas synergistic interactions according to the predefined criterion were confined to the three mixture ratios at 5 µg/mL. At 25 and 50 µg/mL, the mixtures maintained very high absolute antioxidant activity but showed only small positive deviations from Bliss independence. These findings indicate that antioxidant interaction effects in EOB–EOC mixtures are strongly concentration-dependent and should be distinguished from the absolute magnitude of lipid peroxidation inhibition.

#### 2.4.2. ABTS Radical Scavenging Activity

The ABTS^•+^ radical cation decolorization assay is a widely applied method for evaluating antioxidant capacity in complex biological and phytochemical matrices due to its suitability for both hydrophilic and lipophilic antioxidants. The assay reflects mainly single electron transfer (SET) mechanisms, although hydrogen atom transfer (HAT) reactions may also contribute to radical neutralization depending on the chemical structure of antioxidant molecules [55,56].

**Figure 10:**
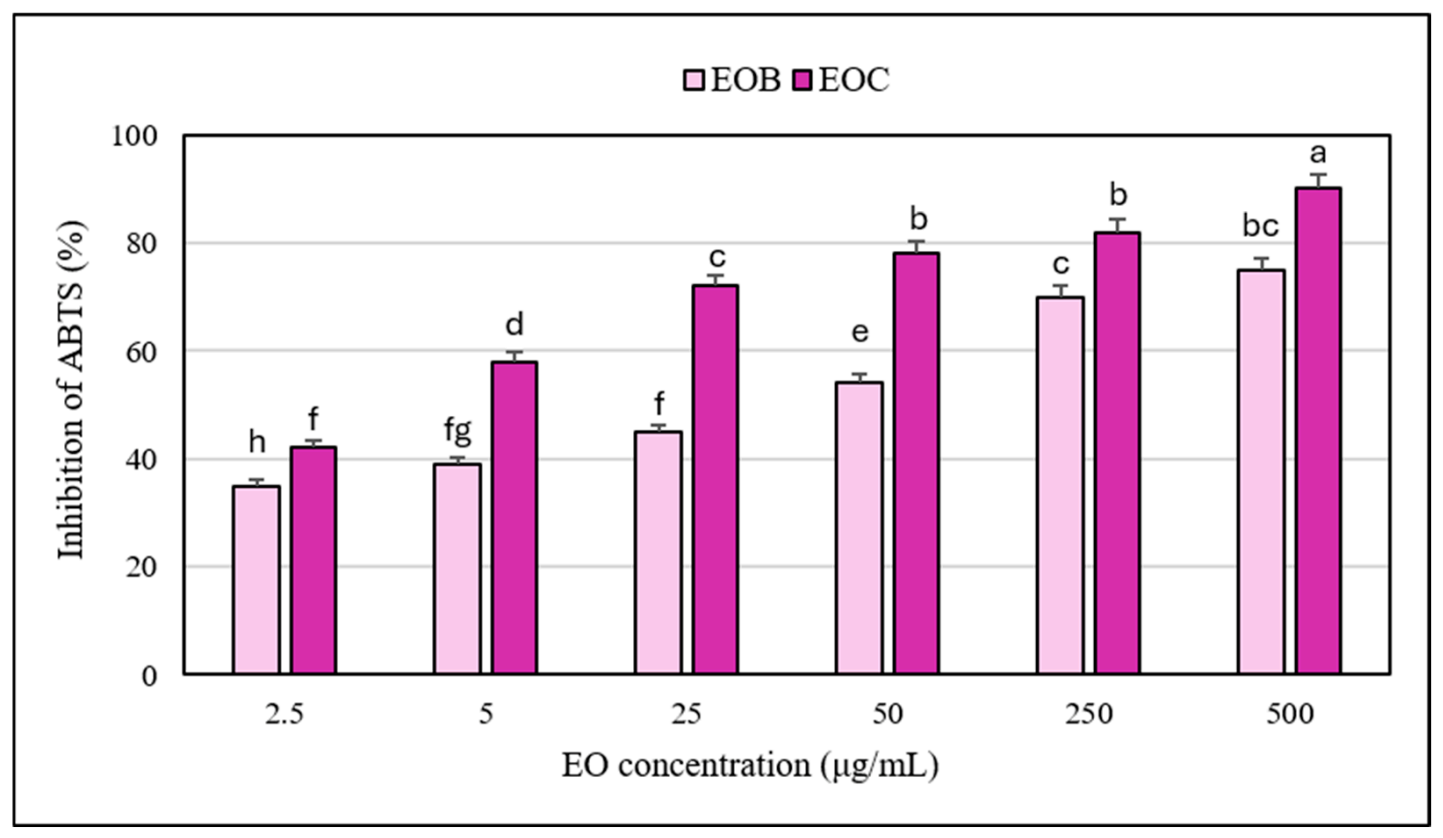
ABTS^•+^ radical scavenging activity of individual basil and clove essential oils. The essential oils obtained from the aerial parts of *Ocimum basilicum* L. (EOB) and the floral buds of *Syzygium aromaticum* L. (EOC) were evaluated at concentrations ranging from 2.5 to 500 µg/mL. Results are expressed as ABTS^•+^ inhibition (%) and presented as mean ± standard deviation (SD). Different letters above the bars indicate statistically significant differences among treatments, according to Tukey’s multiple comparison test (*p* < 0.05).

In the present study, basil (*Ocimum basilicum* L.; EOB) and clove (*Syzygium aromaticum* L.; EOC) essential oils demonstrated a clear concentration-dependent increase in ABTS^•+^ radical scavenging activity across the tested concentration range (2.5–500 µg/mL) (Figure 10). However, substantial differences in antioxidant efficiency were observed between the two essential oils. Clove essential oil consistently exhibited stronger ABTS^•+^ radical scavenging activity than basil essential oil at all evaluated concentrations. The inhibition values increased from 42% to 90% for EOC, whereas EOB showed an increase from 35% to 75%. The higher radical scavenging capacity of EOC was confirmed by IC_50_ analysis. Clove essential oil exhibited an IC_50_ value of 3.75 ± 0.1 µg/mL, whereas basil essential oil showed a markedly higher IC_50_ value of 38.9 ± 0.5 µg/mL. Thus, EOC demonstrated approximately ten-fold greater scavenging potency in the ABTS^•+^ assay compared with EOB.

Ascorbic acid (AsA), used as the reference antioxidant, was evaluated over the concentration range of 0–150 µg/mL and exhibited an IC_50_ value of 9.5 ± 0.3 µg/mL. Accordingly, its ABTS^•+^ radical scavenging potency was intermediate between those of EOC and EOB under the applied experimental conditions. Based on the IC_50_ values, EOC exhibited approximately 2.5-fold greater ABTS^•+^ radical scavenging potency than AsA, whereas AsA was approximately 4.1-fold more potent than EOB. At 150 µg/mL, AsA produced 98% ABTS^•+^ radical scavenging activity, confirming the expected high activity of the reference antioxidant under the applied assay conditions.

Previous studies have also demonstrated ABTS^•+^ radical scavenging activity of both basil and clove essential oils, although considerable variation in the reported IC_50_ values has been observed. Rezzoug et al. reported an ABTS IC_50_ of 0.6870 ± 0.0203 mg/mL (687.0 ± 20.3 µg/mL) for *O. basilicum* essential oil, whereas Nait Irahal et al. reported an IC_50_ of 34.42 µg/mL for *S. aromaticum* bud essential oil [57,58]. The lower IC_50_ values obtained in the present study for EOB (38.9 ± 0.5 µg/mL) and particularly EOC (3.75 ± 0.1 µg/mL) may reflect differences in essential oil chemical composition, chemotype, and experimental conditions, including the sample-to-ABTS ratio and reaction time. Accordingly, direct comparison of absolute IC_50_ values among studies should be interpreted cautiously.

The difference in ABTS^•+^ radical-scavenging potency between EOC and EOB may reflect their distinct overall chemical profiles. Published studies have associated eugenol and other phenolic constituents of essential oils with radical-scavenging activity [16,26,59,60]. However, the contribution of individual constituents to the activity observed in the present study was not determined. Accordingly, the higher ABTS^•+^ radical-scavenging activity of EOC cannot be attributed specifically to eugenol, β-caryophyllene, eugenyl acetate, or any other individual compound and should instead be interpreted at the level of the chemically complex essential oil. Likewise, the lower activity of EOB cannot be assigned specifically to estragole or to the relative abundance of particular constituents. The observed difference should therefore be interpreted at the level of the complete essential oils rather than individual constituents. The present study additionally evaluated binary combinations of EOB and EOC across multiple concentrations and mixture ratios.

**Figure 11:**
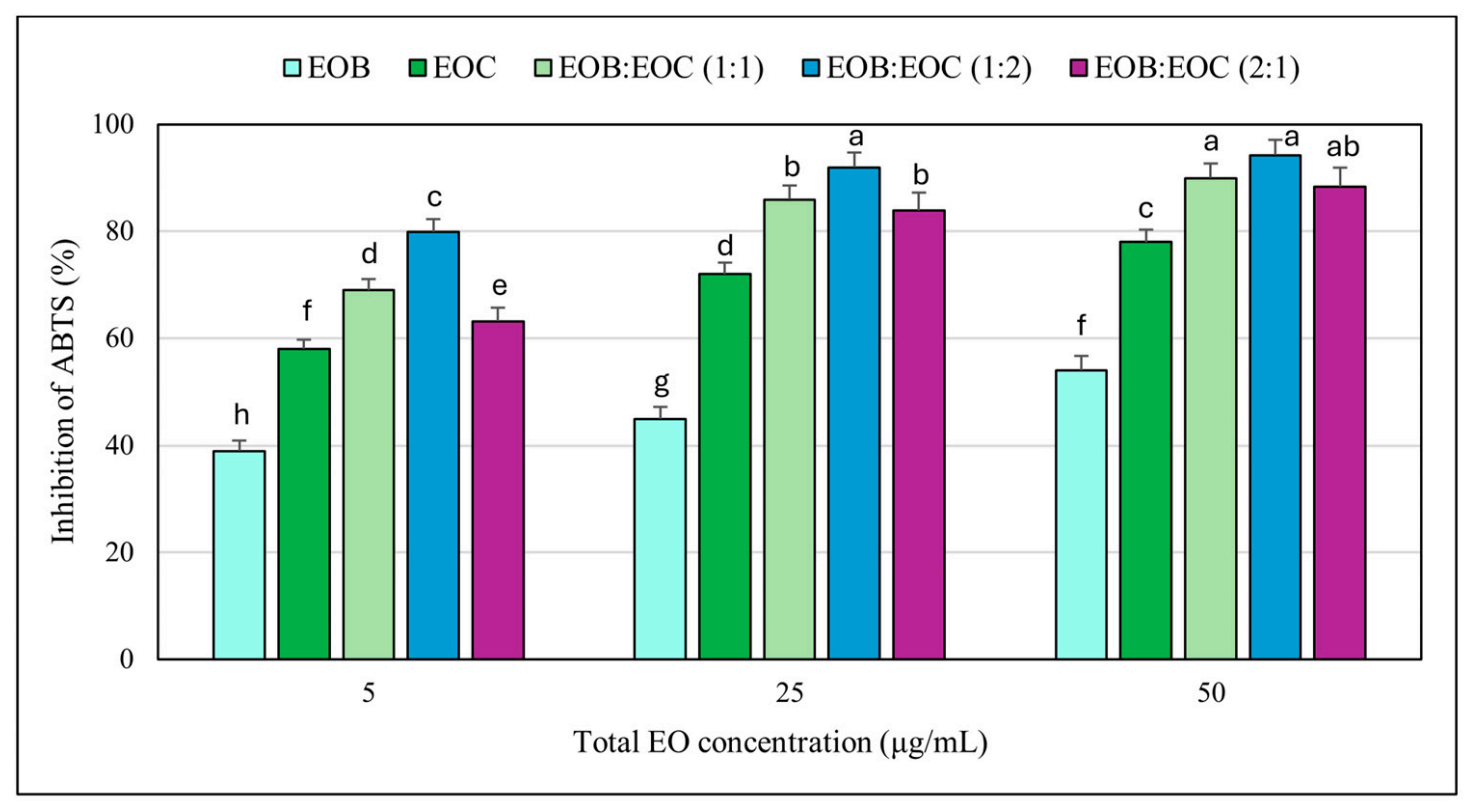
ABTS^•+^ radical scavenging activity of basil (EOB) and clove (EOC) essential oils and their binary mixtures. EOB and EOC were tested individually at concentrations of 5, 25, and 50 µg/mL, while binary EOB:EOC mixtures (1:1, 1:2, and 2:1, *v*/*v*) were evaluated at the same total essential oil concentrations. The previously presented individual EOB and EOC data at 5, 25, and 50 µg/mL are replotted here to facilitate direct comparison with the binary mixtures. Results are expressed as ABTS^•+^ radical scavenging activity (%) and presented as mean ± SD from three independent experiments, each with two technical replicates. The effects of formulation and concentration were evaluated by ANOVA followed by Tukey’s multiple comparison test. Different letters indicate significant differences among all formulation–concentration combinations (*p* < 0.05).

Binary mixtures of EOB and EOC were tested at total essential oil concentrations of 5, 25, and 50 µg/mL using three volumetric ratios (1:1, 1:2, and 2:1, *v*/*v*) (Figure 11). For direct comparison with the binary mixtures, the previously presented individual EOB and EOC data at the corresponding concentrations were replotted in Figure 11. At 5 µg/mL, ABTS^•+^ radical scavenging activity of the mixtures ranged from 63.2% to 79.9%, with the highest activity observed for the 1:2 EOB:EOC mixture. At 25 µg/mL, the corresponding values ranged from 83.9% to 92.0%, whereas at 50 µg/mL the mixtures exhibited 88.4–94.3% activity. At each total essential oil concentration, the 1:2 formulation showed the highest radical scavenging activity among the tested ratios, consistent with the greater activity of EOC observed in the individual-oil analysis.

Interactions between EOB and EOC were further evaluated using the Bliss independence model (Figure 5; Table S4). All ΔBliss values were positive, indicating that the experimentally observed ABTS^•+^ radical scavenging responses of all tested mixtures exceeded the corresponding responses expected under Bliss independence. At 5 µg/mL, ΔBliss values were +6.7, +14.5, and +5.8 percentage points for the 1:1, 1:2, and 2:1 mixtures, respectively. At 25 µg/mL, the corresponding values were +5.6, +10.6, and +5.1 percentage points, whereas at 50 µg/mL they were +5.4, +8.4, and +4.1 percentage points. Only the 1:2 EOB:EOC mixtures at 5 and 25 µg/mL exceeded the predefined +10-percentage-point threshold and were therefore considered consistent with synergistic interactions according to the operational criterion adopted in this study. The remaining combinations showed positive ΔBliss values within the −10 to +10 percentage-point interval and were interpreted as showing no substantial deviation from Bliss independence. No antagonistic interactions were observed under the tested conditions.

The 1:2 formulation, containing the higher proportion of EOC, produced the largest positive deviations from the Bliss-independence expectation at all three concentrations. This pattern is consistent with the substantially greater ABTS^•+^ radical scavenging potency of EOC when tested individually, as reflected by its markedly lower IC_50_ value (3.75 ± 0.1 µg/mL) compared with EOB (38.9 ± 0.5 µg/mL). Nevertheless, the positive ΔBliss values cannot be attributed solely to the greater intrinsic antioxidant activity of EOC, because the Bliss model compares the experimentally observed mixture response with the effect expected from the activities of both individual oils at the component concentrations present in the respective mixture.

Because the activities of isolated constituents and their specific contributions were not examined, the molecular basis of the positive Bliss deviations cannot be established from the present experiments. These deviations should therefore be interpreted as quantitative evidence of interaction at the assay-response level rather than as evidence of complementary actions of specific constituents or defined molecular mechanisms. The decrease in ΔBliss magnitude at 50 µg/mL, despite the high absolute radical-scavenging activity of all mixtures, may partly reflect the approach of the assay response toward higher inhibition levels. Under these conditions, the remaining response range available for additional enhancement becomes progressively restricted. Accordingly, high absolute ABTS^•+^ radical scavenging activity should not itself be regarded as evidence of synergism; rather, within the Bliss framework applied here, synergistic interaction requires a sufficiently large positive deviation from the response expected under independent action.

Overall, EOB–EOC interactions in the ABTS^•+^ assay were concentration- and ratio-dependent rather than uniformly synergistic. These findings emphasize the importance of evaluating essential oil combinations across multiple concentrations and mixture ratios rather than inferring interaction behaviour from a single experimental condition.

### 2.5. Study Limitations

Several limitations of the present study should be acknowledged. First, the biological activities were evaluated using cell-free assays, which do not account for cellular uptake, metabolism, bioavailability, or tissue-level responses. Cytotoxicity was not assessed, and the observed antioxidant and enzyme-inhibitory effects therefore require confirmation in cellular and in vivo models. Second, the interaction analysis was restricted to three EOB:EOC ratios and three total mixture concentrations, and the observed interaction patterns should not be extrapolated beyond the tested experimental design. Third, the present findings apply to the specific chemical profiles of the basil and clove essential oils characterized in this study and may differ for oils representing other chemotypes or sources. The activities of isolated major constituents were not examined, precluding assignment of the observed effects to specific compounds or molecular mechanisms. The concentrations used in the present cell-free assays were selected to characterize concentration–response relationships and mixture interactions under controlled experimental conditions and should not be interpreted as directly applicable concentrations for future topical formulations. Formulation-relevant concentrations would need to be established in cellular and skin-relevant models together with appropriate cytotoxicity and safety assessment.

## 3. Materials and Methods

### 3.1. Plant Material and Essential Oil Isolation

Basil (*Ocimum basilicum* L.) aerial parts and clove (*Syzygium aromaticum* L.) floral buds were purchased from NANGA (Blękwit, Poland). The materials originated from Egypt and Madagascar, respectively (batch nos. 58/N/L/1 and 66/B/O/1). The supplier provided certificates of analysis confirming the botanical identity of both raw materials, together with the results of physicochemical, microbiological, and safety-related testing. Moisture content, total ash, heavy metal concentrations (Pb and Cd), and mycotoxin levels complied with the declared specifications, while *Salmonella* spp. was not detected in either sample. Essential oils were obtained by hydrodistillation of separate 40 g portions of dried basil and clove plant material using a Clevenger-type apparatus [54]. The distillation was carried out for 3 h under atmospheric pressure. The extraction yields were 1.0% (*v*/*w*) for basil essential oil and 1.75% (*v*/*w*) for clove essential oil. The isolated oils were dehydrated over anhydrous sodium sulfate, filtered, transferred to amber glass vials, and stored at 4 °C until further analyses.

### 3.2. Preparation of Essential Oil Solutions

Working solutions were prepared by diluting the essential oils with ethanol to final concentrations of 2.5, 5, 25, 50, 250, and 500 µg/mL (*w*/*v*). The solutions were vortexed before analysis to ensure homogeneity. Binary mixtures of basil (EOB) and clove (EOC) essential oils were prepared at volumetric ratios (*v*/*v*) of 1:1, 1:2, and 2:1. Each mixture ratio was evaluated at total essential oil concentrations of 5, 25, and 50 µg/mL (*w*/*v*), where the total essential oil concentration corresponded to the sum of the concentrations of EOB and EOC in the respective binary formulation. Detailed component concentrations of EOB and EOC corresponding to each mixture ratio and total essential oil concentration are presented in Tables S1–S4. The selected concentration range was chosen to provide a concentration-dependent assessment of the interactions while maintaining an adequate dynamic response range and minimizing the influence of response saturation at higher essential oil concentrations. No turbidity or phase separation was observed in the individual EO solutions or binary mixtures under the applied experimental conditions, and all preparations remained optically clear and homogeneous. All binary mixtures were vortex-mixed immediately before analysis to ensure homogeneity.

### 3.3. GC-MS Characterization of Essential Oils

The volatile constituents of the examined essential oils (EOB and EOC) were identified and quantified using gas chromatography-mass spectrometry (GC-MS), following our previously described procedure with minor modifications [53,54]. GC-MS analyses were performed on a TRACE GC–ULTRA gas chromatograph connected to a Polaris Q ion-trap mass spectrometer (Thermo Fisher Scientific, Waltham, MA, USA). Volatile compounds were separated on an Equity-5 fused-silica capillary column (Supelco, Bellefonte, PA, USA) under chromatographic conditions optimized for the separation of volatile constituents. Mass spectra were obtained under electron impact (EI) ionization at 70 eV. Each compound was identified by comparing its mass spectral fragmentation pattern with the NIST Mass Spectral Library and by matching the calculated retention indices with those found in the literature [61,62]. Compounds that showed consistent spectral matches and retention index values were included in the final chemical composition of the essential oils.

### 3.4. Cyclooxygenase-2 (COX-2) Inhibitory Activity Assay

The cyclooxygenase-2 (COX-2) inhibitory activity of basil (*Ocimum basilicum* L.; EOB) and clove (*Syzygium aromaticum* L.; EOC) essential oils was investigated fluorometrically using the COX-2 Inhibitor Screening Kit (catalogue no. ab283401, Abcam, Cambridge, UK) following the manufacturer’s instructions with slight modifications. The assay is based on the fluorometric detection of prostaglandin G_2_ (PGG_2_), which is an intermediate produced during the cyclooxygenase-catalysed oxidation of arachidonic acid. Celecoxib was used as the reference COX-2 inhibitor. Because the concentration of the celecoxib reagent supplied with the assay kit was not specified, a separately prepared celecoxib solution of defined concentration was used for quantitative reference measurements. To determine its concentration–response relationship and IC_50_ value, celecoxib was evaluated over a concentration range of 0–30 µg/mL. In addition, celecoxib at 25 µg/mL was included as the positive-control point in the assay. Wells containing human recombinant COX-2 enzyme without EO, but with an equivalent volume of ethanol to that introduced with the EO samples, served as the solvent-matched enzyme control (100% COX-2 activity). Following the addition of the reaction mixture and arachidonic acid substrate, fluorescence was recorded using a Varioskan™ LUX multimode microplate reader (Thermo Fisher Scientific, Waltham, MA, USA) in kinetic mode for 10 min. at 25 °C. Fluorescence intensity was recorded at an excitation wavelength of 535 nm and an emission wavelength of 587 nm. Inhibition of COX-2 activity was calculated as follows:

COX-2 inhibition (%) = [(Slope*_EC_* − Slope*_S_*)/Slope*_EC_*] × 100 (1)

where Slope*_EC_* is the reaction rate (ΔRFU × min^−1^) of the enzyme control (100% COX-2 activity), whereas Slope*_S_* is the reaction rate measured in the presence of the tested sample (EO or positive inhibitor control).

The IC_50_ value was defined as the concentration of EO or celecoxib (μg/mL) required to inhibit 50% of COX-2 activity under the experimental conditions.

### 3.5. Collagenase Inhibitory Activity Assay

The inhibitory activity of basil and clove essential oils against collagenase was evaluated fluorometrically using the Collagenase Inhibitor Screening Kit (catalogue no. ab211108, Abcam, Cambridge, UK), following the manufacturer’s protocol with minor modifications. 1,10-Phenanthroline was used as the reference collagenase inhibitor. To determine its concentration–response relationship and IC_50_ value, 1,10-phenanthroline was evaluated over a concentration range of 0–200 µg/mL. In addition, 1,10-phenanthroline at a final assay concentration of 0.8 mM (equivalent to approximately 144.2 µg/mL) was included as the positive-control point according to the manufacturer’s protocol, whereas wells containing collagenase without EO, but with an equivalent volume of ethanol to that introduced with the EO samples, served as the solvent-matched enzyme control (100% collagenase activity). After adding the fluorogenic substrate, fluorescence was recorded using a Varioskan™ LUX multimode microplate reader (Thermo Fisher Scientific, Waltham, MA, USA) in kinetic mode for 60 min. at 37 °C in the dark. Fluorescence intensity was recorded at an excitation wavelength of 490 nm and an emission wavelength of 520 nm. The inhibitory effect against collagenase was expressed as a percentage using the following equation:

Collagenase inhibition (%) = [(RFU*_EC_* − RFU*_S_*)/RFU*_EC_*] × 100 (2)

where RFU*_EC_* is the fluorescence intensity of the untreated enzyme control, representing 100% collagenase activity, and RFU*_S_* is the fluorescence intensity measured in the presence of the tested sample (EO or positive inhibitor control).

IC_50_ values were calculated as the concentration of EO or 1,10-phenanthroline (μg/mL) required to inhibit 50% of collagenase activity under the experimental conditions.

### 3.6. Antioxidant Capacity of the Essential Oils

#### 3.6.1. Inhibition of Linoleic Acid Peroxidation Assay

The antioxidant activity of the tested essential oils was evaluated by measuring their ability to inhibit hemoglobin-induced peroxidation of linoleic acid, using the ferric thiocyanate assay described by Kuo et al. [63], with minor modifications as previously described [54]. The reaction mixture contained 370 μL of 0.05 M phosphate buffer (pH 7.0) with 4 mM linoleic acid and 0.05% (*v*/*v*) Tween 20, to which 10 μL of the tested essential oil was added. Control samples contained an equal volume of ethanol instead of the essential oil. Following equilibration at 37 °C for 3 min., oxidation was initiated by adding 20 μL of freshly prepared 0.035% hemoglobin solution in deionized water. The mixtures were incubated at 37 °C for 10 min. under constant agitation (120 rpm). The reaction was stopped by adding 5 mL of 0.6% hydrochloric acid in ethanol. Lipid hydroperoxide formation was determined using the ferric thiocyanate reaction. After adding 100 μL of 20 mM FeCl_2_ and 100 μL of 30% ammonium thiocyanate, absorbance was recorded at 480 nm. Butylated hydroxytoluene (BHT) was used as the reference antioxidant. To determine its concentration–response relationship and IC_50_ value, BHT was evaluated over a concentration range of 0–100 µg/mL. In addition, BHT at 20 µg/mL was included as a positive-control point in each assay.

The extent of lipid peroxidation inhibition was calculated according to the following equation:

Lipid peroxidation inhibition (%) = [1 − (A_S_ − A_0_)/(A_C_ − A_0_)] × 100 (3)

where A_0_ represents the absorbance of the tested sample in the absence of hemoglobin, A_C_ corresponds to the negative control containing ethanol, and A_S_ denotes the absorbance of the complete reaction mixture containing the tested EO sample or BHT.

IC_50_ values represented the concentration of EO or BHT (μg/mL) required to achieve 50% inhibition of lipid peroxidation.

#### 3.6.2. ABTS^•+^ Radical Scavenging Assay

The radical scavenging activity of the essential oils was assessed using the ABTS^•+^ decolorization assay following the procedure of Re et al. [55] with minor modifications. The ABTS radical cation was generated by mixing 7 mM ABTS with 2.45 mM potassium persulfate at a volume ratio of 1:0.5. The reaction mixture was protected from light and incubated at room temperature for 16 h to ensure complete radical formation. Before analysis, the stock solution was diluted with 96% ethanol to obtain an absorbance of approximately 0.7 at 734 nm. For each measurement, 50 μL of the essential oil solution at the tested concentrations (2.5–500 μg/mL), containing 0.5% (*v*/*v*) Tween 20 was mixed with 350 μL of the freshly prepared ABTS^•+^ working solution. The samples were vortex-mixed for 15 s and incubated for 30 min. at ambient temperature in the absence of light. Absorbance was measured at 734 nm using an Epoch microplate spectrophotometer (BioTek, Winooski, VT, USA). Ascorbic acid (AsA) was used as the reference antioxidant and was evaluated over a concentration range of 0–150 μg/mL for determination of its IC_50_ value. In addition, a freshly prepared AsA solution at 150 μg/mL was analysed in parallel as a single-point positive control. ABTS^•+^ radical scavenging activity was determined using the following equation:

Inhibition (%) = [(A_0_ − A_S_)/A_0_] × 100 (4)

where A_0_ is the absorbance of the untreated ABTS^•+^ solution and A_S_ is the absorbance recorded after incubation with the tested EO sample or the reference antioxidant.

The antioxidant activity of the investigated EOs and AsA was expressed as the IC_50_ value, corresponding to the concentration (μg/mL) required to scavenge 50% of ABTS^•+^ radicals.

### 3.7. Bliss Independence Analysis of Basil–Clove Essential Oil Interactions

Interactions between basil and clove essential oils were evaluated using the Bliss independence model [19,20]. The Bliss model was applied as an effect-based reference framework to determine whether the experimentally observed response of each binary EOB:EOC combination differed from the response expected under independent action of the two essential oils. Bliss independence has also been applied previously for the quantitative assessment of interactions involving plant oil mixtures [21].

Binary EOB:EOC mixtures were investigated at three volumetric ratios (1:1, 1:2, and 2:1; *v*/*v*) and at total essential oil concentrations of 5, 25, and 50 µg/mL. Thus, nine combination conditions were evaluated for each assay. The selected concentration range was chosen to provide a concentration-dependent assessment of the interactions while maintaining an adequate dynamic response range and minimizing the influence of response saturation at higher essential oil concentrations.

For each combination condition, the expected response according to the Bliss independence model (*E_Bliss_*) was calculated as:

(5) $$E_{\mathrm{Bliss}}=E_{A}+E_{B}-\;\frac{E_{A}\;E_{B}}{100}$$

where *E_A_* and *E_B_* represent the percentage effects of EOB and EOC, respectively, when tested individually at the concentrations corresponding to their respective contributions to the binary mixture. The individual effects of EOB and EOC at these component concentrations were determined experimentally under the same assay conditions as the corresponding binary mixtures.

The deviation of the experimentally observed mixture response from the Bliss-expected response was expressed as excess over Bliss (ΔBliss):

ΔBliss = *E*_observed_ − *E_Bliss_* (6)

where *E*_observed_ represents the experimentally determined percentage response of the respective binary mixture. Accordingly, ΔBliss values were expressed in percentage points. Positive ΔBliss values indicate responses greater than predicted under Bliss independence, whereas negative values indicate responses lower than the Bliss-expected response. For descriptive interpretation of interaction magnitude, an operational threshold of ±10 percentage points was adopted, analogous to the heuristic interpretation of excess-response synergy scores used in the SynergyFinder framework [64,65]. Interaction outcomes were classified as follows: ΔBliss > +10, consistent with a synergistic interaction; −10 ≤ ΔBliss ≤ +10, showing no substantial deviation from Bliss independence; ΔBliss < −10, consistent with an antagonistic interaction. This threshold was used solely as a descriptive effect-size criterion and not as a test of statistical significance.

Bliss interaction analysis was performed separately for COX-2 inhibition, collagenase inhibition, linoleic acid peroxidation inhibition, and ABTS^•+^ radical scavenging activity. For the Bliss analysis, the resulting EOB, EOC, and corresponding binary-mixture responses were matched within the same independent experiment, and *E_Bliss_* and ΔBliss were calculated separately for each of the three independent experiments. The resulting ΔBliss values were subsequently summarized as mean ± standard deviation (SD). The ΔBliss heatmap was generated using Python version 3.13.15 [66]. Data processing and visualization were performed using NumPy (version 2.5.2) and matplotlib (version 3.11.1).

### 3.8. Statistical Analysis

Each assay was performed in three independent experiments, with two technical replicates per experiment. Technical replicates were averaged within each independent experiment, and the resulting independent values were used for statistical analysis. Results are presented as mean ± standard deviation (SD). Statistical differences among the investigated EOs and their binary mixtures were assessed using analysis of variance (ANOVA) followed by Tukey’s multiple comparison test, with *p* < 0.05 considered statistically significant. For the Bliss interaction analysis, *E_Bliss_* and ΔBliss were calculated separately for each independent experiment, and the resulting ΔBliss values are presented as mean ± SD from three independent experiments. Statistical analyses were carried out using Statistica 13.3 software (StatSoft, Kraków, Poland).

## 4. Conclusions

The combined GC-MS analysis and complementary in vitro assays revealed distinct activity profiles of the investigated essential oils. EOC showed greater ABTS^•+^ radical-scavenging activity and stronger inhibition of COX-2 and collagenase, whereas EOB showed greater inhibition of linoleic acid peroxidation. Binary-mixture effects were dependent on both concentration and mixture ratio. According to the predefined Bliss criterion, interactions consistent with synergy were observed only under selected experimental conditions, while the remaining combinations showed no substantial deviation from Bliss independence; no antagonistic interactions were detected. The most active EOB:EOC ratio among those tested depended on the assay endpoint. Because isolated constituents were not examined, the observed activities and interaction patterns should be attributed to the essential oils as complex mixtures rather than to individual compounds or specific molecular mechanisms. These cell-free findings warrant further evaluation in cellular and in vivo models, together with appropriate assessment of efficacy and safety.

## Figures and Tables

**Table 1:** Volatile composition of the basil essential oil (EOB) determined by GC-MS analysis.

Chemical Constituents	RT	RI	Peak Area (%)
*α*-Pinene	11.02	931	0.2
*β*-Pinene	12.66	974	0.1
*β*-Myrcene	13.30	991	<0.1
Δ2-Carene	13.72	1002	<0.1
*p*-Cymene	14.53	1023	0.1
Limonene	14.71	1027	0.1
1,8-Cineole	14.78	1029	1.9
*γ*-Terpinene	15.88	1058	0.1
(*Z*)-Sabinene hydrate	16.19	1066	<0.1
Fenchone	16.99	1087	0.1
Linalool	17.47	1099	1.6
*α*-Fenchol	17.96	1112	0.2
Camphor	19.09	1143	0.2
Borneol	19.92	1166	0.1
Terpinen-4-ol	20.34	1177	0.3
*α*-Terpineol	20.84	1190	0.3
Estragole	21.20	1198	73.0
*trans*-Anethole	24.21	1286	0.5
*α*-Copaene	27.27	1377	0.1
*β*-Bourbonene	27.57	1386	0.1
*β*-Elemene	27.80	1393	0.2
Methyl eugenol	28.17	1404	7.2
(*E*)-*β*-Caryophyllene	28.71	1421	3.6
(*E*)-*α*-Bergamotene	29.19	1437	1.8
*α*-Guaiene	29.26	1440	0.1
Guaia-6,9-diene	29.39	1444	0.1
*α*-Humulene	29.79	1456	1.4
*cis*-Muurola-4(14),5-diene	30.06	1465	0.1
Germacrene D	30.64	1483	0.9
(*E*)-*β*-Ionone	30.72	1486	0.2
*β*-Selinene	30.81	1489	0.1
*α*-Zingiberene	31.07	1497	0.2
*α*-Bulnesene	31.44	1508	0.2
*γ*-Cadinene	31.64	1517	0.6
*δ*-Cadinene	31.91	1526	0.2
(*E*)-*α*-Bisabolene	32.47	1545	1.4
Spathulenol	33.55	1581	0.1
Caryophyllene oxide	33.72	1586	0.5
Humulene epoxide II	34.48	1613	0.2
1,10-di-*epi*-Cubenol	34.63	1618	0.1
*epi*-*α*-Cadinol	35.36	1644	0.8
**Total**	-	-	**99.2**

GC-MS—gas chromatography-mass spectrometry; RT—retention time (min.); RI—retention index.

**Table 2:** Volatile composition of the clove essential oil (EOC) determined by GC-MS analysis.

Chemical Constituents	RT	RI	Peak Area (%)
4-Methylhexanol	15.28	1042	0.1
(*E*)-*β*-Ocimene	15.47	1047	<0.1
2-Nonanone	17.16	1091	<0.1
Linalool	17.46	1099	0.1
Benzyl acetate	19.84	1163	<0.1
Ethyl benzoate	20.07	1170	<0.1
Methyl salicylate	20.93	1193	0.1
Estragole	21.20	1198	<0.1
Chavicol	23.08	1254	<0.1
*trans*-Anethole	24.21	1286	0.1
*α*-Cubebene	26.38	1351	0.1
Eugenol	26.62	1358	55.0
*α*-Ylangene	27.12	1373	<0.1
*α*-Copaene	27.27	1377	0.9
*β*-Elemene	27.80	1393	<0.1
(*E*)-*β*-Caryophyllene	28.70	1421	32.9
*β*-Copaene	28.74	1423	<0.1
*α*-Humulene	29.78	1456	3.5
*γ*-Muurolene	30.48	1479	0.2
Germacrene D	30.63	1483	0.3
*β*-Selinene	30.81	1489	<0.1
*α*-Zingiberene	31.05	1497	0.1
*α*-Muurolene	31.20	1502	0.1
(*E*,*E*)-*α*-Farnesene	31.39	1508	0.4
*γ*-Cadinene	31.64	1517	0.1
*δ*-Cadinene	31.92	1526	0.5
Eugenyl acetate	31.94	1527	4.7
*α*-Cadinene	32.33	1540	<0.1
Caryophyllene oxide	33.71	1586	0.2
**Total**	-	-	**99.8**

GC-MS—gas chromatography-mass spectrometry; RT—retention time (min.); RI—retention index.

## Data Availability

The data presented in the study are available in the article.

## Supplementary Materials

The following supporting information can be downloaded at https://www.mdpi.com/article/10.3390/molecules31183292/s1; Table S1: Bliss independence analysis of interactions between basil (EOB) and clove (EOC) essential oils for cyclooxygenase-2 (COX-2) inhibition; Table S2: Bliss independence analysis of interactions between basil (EOB) and clove (EOC) essential oils for collagenase inhibition; Table S3: Bliss independence analysis of interactions between basil (EOB) and clove (EOC) essential oils for linoleic acid peroxidation inhibition; Table S4: Bliss independence analysis of interactions between basil (EOB) and clove (EOC) essential oils for ABTS^•+^ radical scavenging activity.
