# Supplementary material for: GC-MS Profiling, Antioxidant and Enzyme Inhibitory Activities of Basil (Ocimum basilicum L.) and Clove (Syzygium aromaticum L.) Essential Oils and Their Binary Mixtures
Source: Molecules. 2026 Sep 17;31(18):3292. doi: 10.3390/molecules31183292 (PMC13609182; doi:10.3390/molecules31183292)
Supplement: Supplementary file 1 [file molecules-31-03292-s001.zip › Table S1.pdf]

Table S1. Bliss independence analysis of interactions between basil (EOB) and clove (EOC) essential oils for cyclooxygenase-2 (COX-2) inhibition.

**Total EO concentration (µg/mL)**	**EOB:EOC ratio (v/v)**	**EOB concentration (µg/mL)**	**EOB effect, E_A (%)**	**EOC concentration (µg/mL)**	**EOC effect, E_B (%)**	**E_{Bliss} (%)**	**Observed mixture effect, $E_{observed}$ (%)**	**ΔBliss (percentage points)**
5	1:1	2.50	28.0 ± 1.0	2.50	36.0 ± 2.0	53.9 ± 2.9	59.0 ± 2.2	+5.1 ± 0.4
5	1:2	1.67	23.9 ± 0.9	3.33	41.4 ± 2.3	55.4 ± 2.7	69.0 ± 1.8	**+13.6 ± 0.9**
5	2:1	3.33	30.9 ± 1.5	1.67	28.4 ± 1.1	50.5 ± 2.5	54.9 ± 1.5	+4.4 ± 0.3
25	1:1	12.50	41.8 ± 1.8	12.50	51.3 ± 2.5	71.7 ± 3.2	77.0 ± 4.6	+5.3 ± 0.2
25	1:2	8.33	38.8 ± 2.1	16.67	52.0 ± 2.9	70.6 ± 3.6	83.5 ± 5.1	**+12.9 ± 0.7**
25	2:1	16.67	44.0 ± 1.9	8.33	50.3 ± 1.3	72.2 ± 4.2	75.0 ± 1.6	+2.8 ± 0.3
50	1:1	25.00	47.0 ± 2.7	25.00	53.0 ± 3.5	75.1 ± 3.8	81.6 ± 1.7	+6.5 ± 0.4
50	1:2	16.67	44.3 ± 2.5	33.33	58.0 ± 4.4	76.6 ± 5.2	85.1 ± 6.1	+8.5 ± 0.6
50	2:1	33.33	49.1 ± 3.2	16.67	52.7 ± 3.7	75.9 ± 4.9	79.3 ± 3.7	+3.4 ± 0.2

Values are presented as mean ± SD from three independent experiments. EOB–basil essential oil; EOC–clove essential oil; E_A and E_B–percentage effects of EOB and EOC, respectively, when tested individually at concentrations corresponding to their contributions to the respective binary mixture; E_{Bliss}, effect expected according to the Bliss independence model, calculated as $E_{\text{Bliss}} = E_A + E_B - (E_A \times E_B/100)$; E_{observed}, experimentally observed percentage effect of the binary EOB:EOC mixture; ΔBliss, excess over Bliss, calculated as $\Delta\text{Bliss} = E_{\text{observed}} - E_{\text{Bliss}}$ and expressed in percentage points. For descriptive interpretation, $\Delta\text{Bliss} > +10$ percentage points was considered consistent with a synergistic interaction, $-10 \leq \Delta\text{Bliss} \leq +10$ percentage points indicated no substantial deviation from Bliss independence, and $\Delta\text{Bliss} < -10$ percentage points was considered consistent with an antagonistic interaction.
