# Supplementary material for: GC-MS Profiling, Antioxidant and Enzyme Inhibitory Activities of Basil (Ocimum basilicum L.) and Clove (Syzygium aromaticum L.) Essential Oils and Their Binary Mixtures
Source: Molecules. 2026 Sep 17;31(18):3292. doi: 10.3390/molecules31183292 (PMC13609182; doi:10.3390/molecules31183292)
Supplement: Supplementary file 1 [file molecules-31-03292-s001.zip › Table S2.pdf]

Table S2. Bliss independence analysis of interactions between basil (EOB) and clove (EOC) essential oils for collagenase inhibition.

**Total EO concentration (µg/mL)**	**EOB:EOC ratio (v/v)**	**EOB concentration (µg/mL)**	**EOB effect, E_A (%)**	**EOC concentration (µg/mL)**	**EOC effect, E_B (%)**	**E_{Bliss} (%)**	**Observed mixture effect, $E_{observed}$ (%)**	**ΔBliss (percentage points)**
5	1:1	2.50	5.0 ± 0.2	2.50	24.0 ± 1.0	27.8 ± 1.2	32.0 ± 1.6	+4.2 ± 0.2
5	1:2	1.67	3.3 ± 0.5	3.33	26.1 ± 1.2	28.5 ± 1.4	41.0 ± 2.9	**+12.5 ± 0.9**
5	2:1	3.33	6.2 ± 0.8	1.67	21.1 ± 0.9	26.0 ± 1.2	29.8 ± 1.4	+3.8 ± 0.2
25	1:1	12.50	21.7 ± 1.9	12.50	33.0 ± 1.5	47.5 ± 2.5	53.0 ± 3.1	+5.5 ± 0.3
25	1:2	8.33	15.6 ± 1.0	16.67	34.2 ± 1.7	44.5 ± 1.9	56.0 ± 3.5	**+11.5 ± 0.8**
25	2:1	16.67	26.0 ± 1.2	8.33	31.2 ± 1.3	49.1 ± 2.0	53.8 ± 3.2	+4.7 ± 0.2
50	1:1	25.00	32.0 ± 1.4	25.00	36.0 ± 1.8	56.5 ± 2.3	61.8 ± 2.6	+5.3 ± 0.3
50	1:2	16.67	26.5 ± 1.2	33.33	41.4 ± 2.2	56.9 ± 2.8	65.0 ± 3.8	+8.1 ± 0.6
50	2:1	33.33	33.5 ± 1.3	16.67	33.2 ± 1.5	55.6 ± 3.5	60.5 ± 3.3	+4.9 ± 0.4

Values are presented as mean ± SD from three independent experiments. EOB–basil essential oil; EOC–clove essential oil; E_A and E_B–percentage effects of EOB and EOC, respectively, when tested individually at concentrations corresponding to their contributions to the respective binary mixture; E_{Bliss}–effect expected according to the Bliss independence model, calculated as $E_{Bliss} = E_A + E_B - (E_A \times E_B/100)$; $E_{observed}$–experimentally observed percentage effect of the binary EOB:EOC mixture; ΔBliss–the excess over Bliss, calculated as $\Delta Bliss = E_{observed} - E_{Bliss}$ and expressed in percentage points. For descriptive interpretation, $\Delta Bliss > +10$ percentage points was considered consistent with a synergistic interaction, $-10 \leq \Delta Bliss \leq +10$ percentage points indicated no substantial deviation from Bliss independence, and $\Delta Bliss < -10$ percentage points was considered consistent with an antagonistic interaction.
