# Supplementary material for: GC-MS Profiling, Antioxidant and Enzyme Inhibitory Activities of Basil (Ocimum basilicum L.) and Clove (Syzygium aromaticum L.) Essential Oils and Their Binary Mixtures
Source: Molecules. 2026 Sep 17;31(18):3292. doi: 10.3390/molecules31183292 (PMC13609182; doi:10.3390/molecules31183292)
Supplement: Supplementary file 1 [file molecules-31-03292-s001.zip › Table S3.pdf]

Table S3. Bliss independence analysis of interactions between basil (EOB) and clove (EOC) essential oils for linoleic acid peroxidation inhibition.

| **Total EO concentration (µg/mL)** | **EOB:EOC ratio (v/v)** | **EOB concentration (µg/mL)** | **EOB effect, $E_A$ (%)** | **EOC concentration (µg/mL)** | **EOC effect, $E_B$ (%)** | **$E_{Bliss}$ (%)** | **Observed mixture effect, $E_{observed}$ (%)** | **ΔBliss (percentage points)** |
|---|---|---|---|---|---|---|---|---|
| 5 | 1:1 | 2.50 | 44.0 ± 2.1 | 2.50 | 28.0 ± 1.5 | 59.7 ± 1.9 | 76.5 ± 2.6 | **+16.8 ± 1.3** |
| 5 | 1:2 | 1.67 | 21.9 ± 1.0 | 3.33 | 31.7 ± 1.9 | 46.7 ± 1.5 | 64.7 ± 3.8 | **+18.0 ± 2.0** |
| 5 | 2:1 | 3.33 | 59.7 ± 3.2 | 1.67 | 22.8 ± 0.8 | 68.9 ± 2.7 | 88.1 ± 2.6 | **+19.2 ± 1.7** |
| 25 | 1:1 | 12.50 | 86.5 ± 4.7 | 12.50 | 60.6 ± 3.7 | 94.7 ± 4.1 | 97.0 ± 5.7 | +2.3 ± 0.6 |
| 25 | 1:2 | 8.33 | 84.6 ± 3.4 | 16.67 | 68.1 ± 4.5 | 95.1 ± 5.8 | 97.8 ± 6.0 | +2.7 ± 0.8 |
| 25 | 2:1 | 16.67 | 88.0 ± 3.6 | 8.33 | 50.2 ± 1.3 | 94.0 ± 5.1 | 96.9 ± 3.4 | +2.9 ± 0.5 |
| 50 | 1:1 | 25.00 | 90.0 ± 5.5 | 25.00 | 78.5 ± 4.6 | 97.9 ± 4.4 | 99.1 ± 4.5 | +1.2 ± 0.1 |
| 50 | 1:2 | 16.67 | 86.2 ± 4.4 | 33.33 | 76.9 ± 5.5 | 96.8 ± 4.2 | 98.2 ± 4.8 | +1.4 ± 0.2 |
| 50 | 2:1 | 33.33 | 91.9 ± 3.2 | 16.67 | 67.7 ± 4.4 | 97.4 ± 3.5 | 99.4 ± 3.5 | +2.0 ± 0.5 |

Values are presented as mean ± SD from three independent experiments. EOB–basil essential oil; EOC–clove essential oil; $E_A$ and $E_B$–percentage effects of EOB and EOC, respectively, when tested individually at concentrations corresponding to their contributions to the respective binary mixture; $E_{Bliss}$–effect expected according to the Bliss independence model, calculated as $E_{Bliss} = E_A + E_B - (E_A \times E_B/100)$; $E_{observed}$–experimentally observed percentage effect of the binary EOB:EOC mixture; ΔBliss–the excess over Bliss, calculated as $\Delta Bliss = E_{observed} - E_{Bliss}$ and expressed in percentage points. For descriptive interpretation, $\Delta Bliss > +10$ percentage points was considered consistent with a synergistic interaction, $-10 \leq \Delta Bliss \leq +10$ percentage points indicated no substantial deviation from Bliss independence, and $\Delta Bliss < -10$ percentage points was considered consistent with an antagonistic interaction.
