# Supplementary material for: GC-MS Profiling, Antioxidant and Enzyme Inhibitory Activities of Basil (Ocimum basilicum L.) and Clove (Syzygium aromaticum L.) Essential Oils and Their Binary Mixtures
Source: Molecules. 2026 Sep 17;31(18):3292. doi: 10.3390/molecules31183292 (PMC13609182; doi:10.3390/molecules31183292)
Supplement: Supplementary file 1 [file molecules-31-03292-s001.zip › Table S4.pdf]

Table S4. Bliss independence analysis of interactions between basil (EOB) and clove (EOC) essential oils for $ABTS^{•+}$ radical scavenging activity.

**Total EO concentration (µg/mL)**	**EOB:EOC ratio (v/v)**	**EOB concentration (µg/mL)**	**EOB effect, E_A (%)**	**EOC concentration (µg/mL)**	**EOC effect, E_B (%)**	**E_{Bliss} (%)**	**Observed mixture effect, $E_{observed}$ (%)**	**ΔBliss (percentage points)**
5	1:1	2.50	35.0 ± 1.0	2.50	42.0 ± 2.6	62.3 ± 2.1	69.0 ± 1.2	+6.7 ± 0.5
5	1:2	1.67	32.7 ± 0.8	3.33	48.6 ± 2.9	65.4 ± 3.6	79.9 ± 2.5	**+14.5 ± 0.8**
5	2:1	3.33	36.7 ± 1.6	1.67	32.7 ± 1.8	57.4 ± 2.7	63.2 ± 1.4	+5.8 ± 0.6
25	1:1	12.50	42.4 ± 1.7	12.50	66.0 ± 3.4	80.4 ± 0.8	86.0 ± 1.9	+5.6 ± 0.3
25	1:2	8.33	40.9 ± 1.2	16.67	68.5 ± 3.9	81.4 ± 3.9	92.0 ± 2.8	**+10.6 ± 0.5**
25	2:1	16.67	43.5 ± 2.3	8.33	62.4 ± 3.0	78.8 ± 2.9	83.9 ± 1.6	+5.1 ± 0.6
50	1:1	25.00	45.0 ± 1.5	25.00	72.0 ± 4.5	84.6 ± 3.3	90.0 ± 2.9	+5.4 ± 0.4
50	1:2	16.67	44.9 ± 1.9	33.33	74.5 ± 4.1	85.9 ± 0.9	94.3 ± 3.5	+8.4 ± 0.5
50	2:1	33.33	48.7 ± 2.5	16.67	69.4 ± 2.8	84.3 ± 1.5	88.4 ± 1.4	+4.1 ± 0.2

Values are presented as mean ± SD from three independent experiments. EOB–basil essential oil; EOC–clove essential oil; E_A and E_B–percentage effects of EOB and EOC, respectively, when tested individually at concentrations corresponding to their contributions to the respective binary mixture; E_{Bliss}–effect expected according to the Bliss independence model, calculated as $E_{Bliss} = E_A + E_B - (E_A \times E_B/100)$; $E_{observed}$–experimentally observed percentage effect of the binary EOB:EOC mixture; ΔBliss–the excess over Bliss, calculated as $\Delta Bliss = E_{observed} - E_{Bliss}$ and expressed in percentage points. For descriptive interpretation, $\Delta Bliss > +10$ percentage points was considered consistent with a synergistic interaction, $-10 \leq \Delta Bliss \leq +10$ percentage points indicated no substantial deviation from Bliss independence, and $\Delta Bliss < -10$ percentage points was considered consistent with an antagonistic interaction.
